# Supplementary material for: Chromosome arm aneuploidies shape tumour evolution and drug response
Source: Nat Commun. 2020 Jan 23;11:449. doi: 10.1038/s41467-020-14286-0 (PMC6978319; doi:10.1038/s41467-020-14286-0)
Supplement: Supplementary file 1 — Supplementary Information [file 41467_2020_14286_MOESM1_ESM.pdf]

Supplementary Information

Chromosome arm aneuploidies shape tumour evolution, cancer prognosis and drug response

Ankit Shukla[#], Thu H.M. Nguyen[#], Sarat B. Moka, Jonathan J. Ellis, John P. Grady, Harald Oey, Alexandre S. Cristino, Kum Kum Khanna, Dirk P. Kroese, Lutz Krause, Eloise Dray, J. Lynn Fink, Pascal H.G. Duijf*

Index

Figure	Title	Page(s)
**Supplementary Figure 1**	Technical and biological validation of CAA frequencies	2
**Supplementary Figure 2**	CAA frequencies in samples with and without whole-genome doubling	3
**Supplementary Figure 3**	Fractions of tumours with more chromosome arm gains than losses as a function of CAA burden and whole-genome doubling status	4
**Supplementary Figure 4**	CAA burden and intra-tumour chromosome arm gain:loss ratios in all samples and in samples with and without whole-genome doubling	5-6
**Supplementary Figure 5**	CAA burden in primary and metastatic cancers	7-8
**Supplementary Figure 6**	CAA burden in primary and metastatic cancers with and without whole-genome doubling	9-11
**Supplementary Figure 7**	Stochastic tumour evolution modelling	11
**Supplementary Figure 8**	Fractions of patients with significant good and poor survival-predicting CAAs	11
**Supplementary Figure 9**	Networks and matrices of CAA co-occurrences in 31 types of cancer	12-27
**Supplementary Figure 10**	Volcano plots of CAA co-occurrences in 31 types of cancer	28-32
**Supplementary Figure 11**	Pan-cancer survival analysis based on significantly co-occurring CAAs	33
**Supplementary Figure 12**	Performance of CAA- and CFE-based machine learning models	34
**Supplementary References**		35

a

Panel	Axis	Cohort	Samples (n)	Platform	Pipeline/method
b-e	y	TCGA	1094	SNP6 array	This paper
b	x	TCGA	1048	SNP6 array	Taylor *et al.*, 2018
c	x	TCGA	1081	WG microarray	TCGA firehose
d	x	METABRIC	1992	SNP6 array	This paper
e	x	ICGC/PCAWG	214	WGS	PCAWG-11 consensus

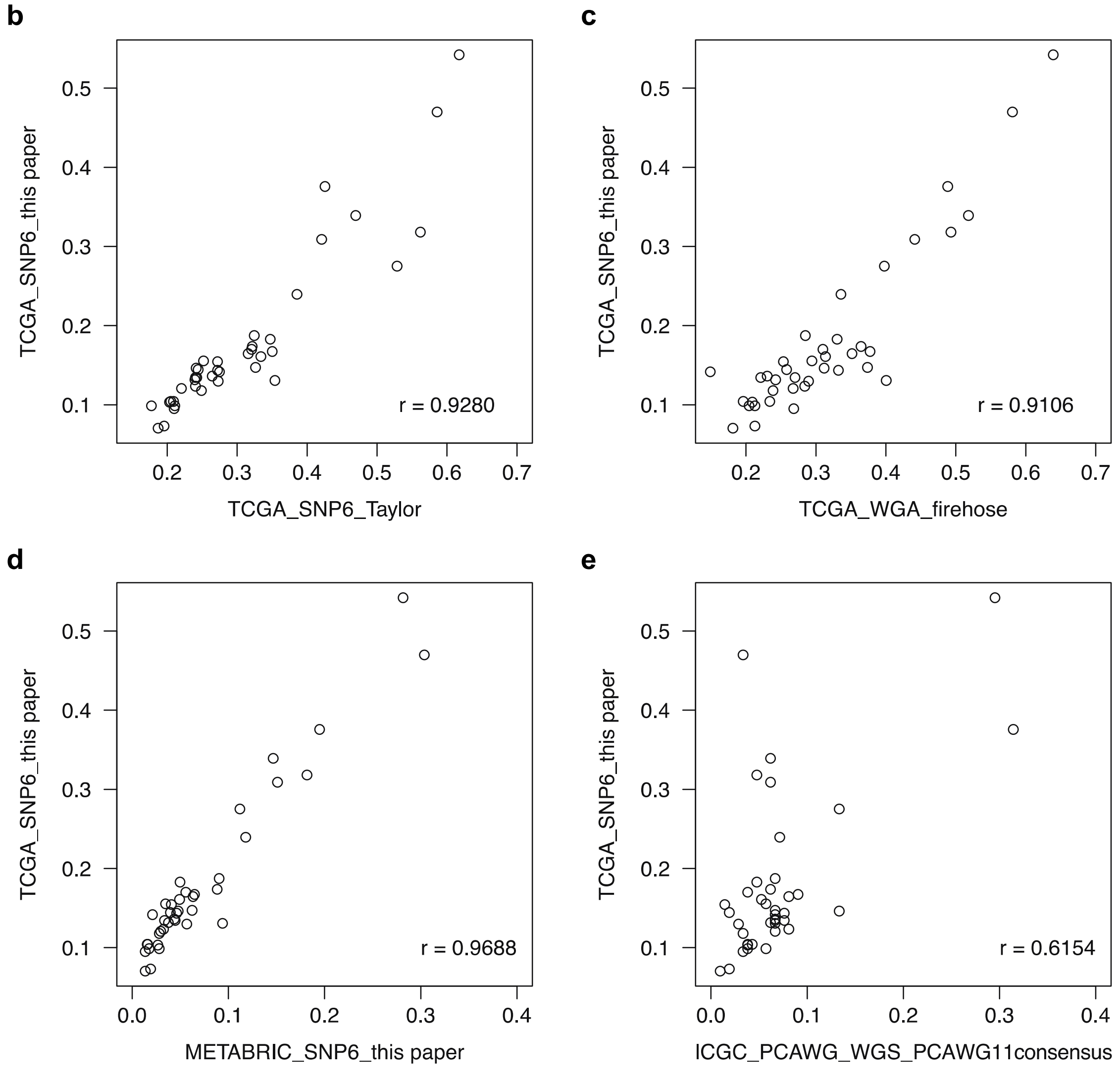

Supplementary Figure 1. Technical and biological validation of CAA frequencies
(a) Table summarizing which patient cohorts, platforms and pipelines were used for validation of breast cancer chromosome arm-level aneuploidy (CAA) frequencies in panels b-e. **(b-e)** Scatter plots comparing the frequencies of each CAA, as determined in this paper, compared to other platforms and methods, including determined by Taylor *et al.*, 2018 [1] and applying our pipeline to the METABRIC dataset [2]. The frequencies of the corresponding CAAs were plotted on the x- and y-axes, as listed in panel (a). Axis label format: <cohort>_<platform>_<pipeline>. Pearson correlation coefficients are indicated. Abbreviations: SNP6 array: single nucleotide polymorphism 6 array; WG array: whole-genome array; WGS: whole genome sequencing.

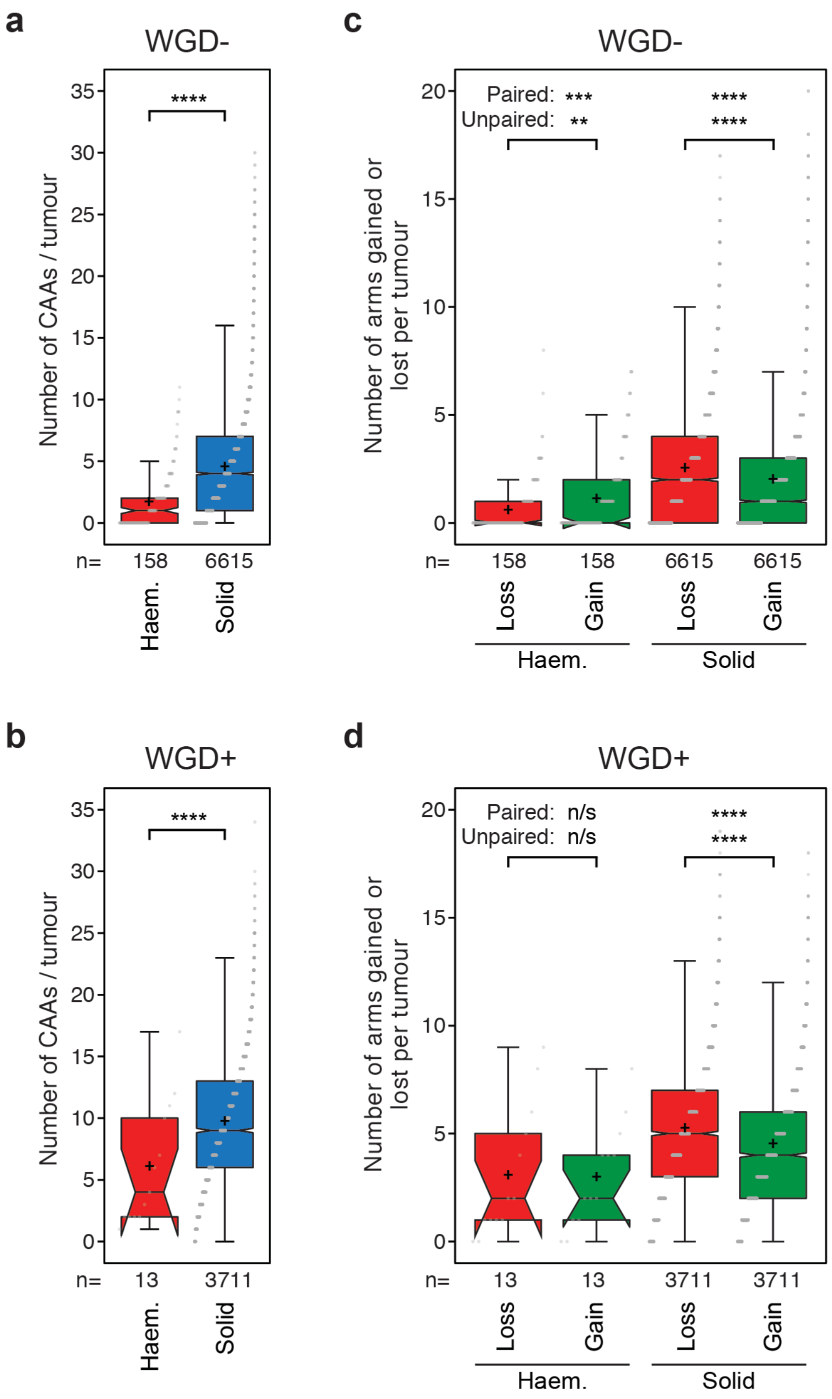

Supplementary Figure 2. CAA frequencies in samples with and without whole-genome doubling
(a, b) Tukey boxplots of CAA burden in haematological (haem.) and solid cancers without whole-genome doubling (WGD-) (a) and with whole-genome doubling (WGD+) (b). Shown are the means ("+"), medians with 95% confidence intervals (lines and notches), interquartile ranges and all data points. The significance levels are $p = 1.6 \times 10^{-21}$ and $p = 0.0055$, respectively, as determined by Mann-Whitney U tests. **(c, d)** Box plots as in (a, b) showing the numbers of chromosome arms lost or gained in haematological and solid cancers without WGD (c) and with WGD (d). The significance levels are: WGD-: haematological paired $p = 0.0002$, unpaired $p = 0.0062$; solid paired $p = 6.8 \times 10^{-72}$, unpaired $p = 2.8 \times 10^{-26}$; WGD+: haematological paired $p = 0.4294$, unpaired $p = 0.4288$; solid paired $p = 1.0 \times 10^{-40}$, unpaired $p = 3.5 \times 10^{-21}$. Paired: Wilcoxon signed-rank tests. Unpaired: Mann-Whitney U tests.

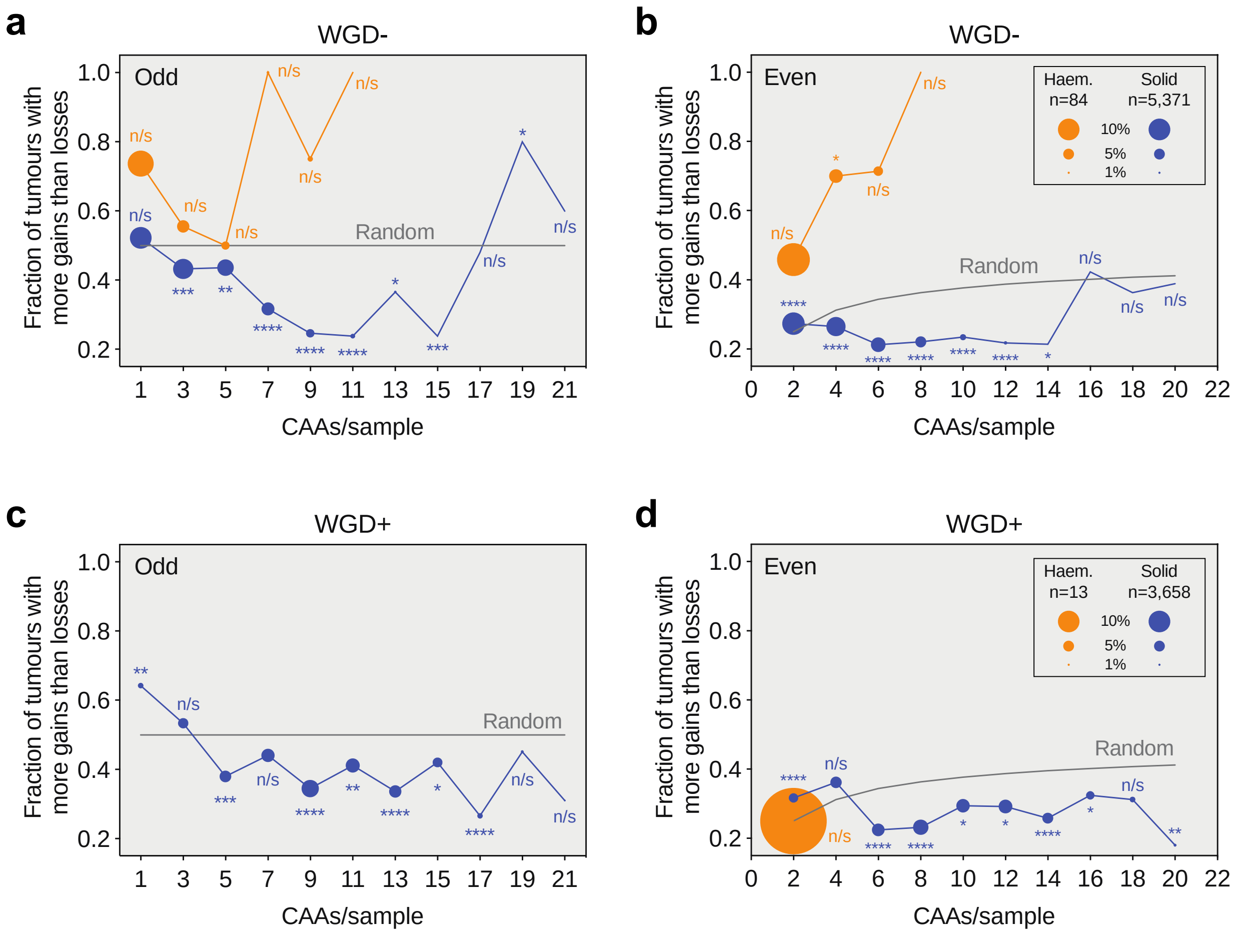

Supplementary Figure 3. Fractions of tumours with more chromosome arm gains than losses as a function of CAA burden and whole-genome doubling status

(a-d) Shooting star plots, as in main Figure 1e, showing fractions of tumours with more chromosome arm gains than losses (*G*>*L*) as a function of the total number of CAAs per sample. Samples shown in main Figure 1e were split according their whole-genome doubling (WGD) status. WGD- and WGD+ samples are shown in panels (a, b) and (c, d), respectively. Odd and even numbers of CAAs per sample are shown separately in panels (a, c) and (b, d), respectively. Dot sizes are proportional to the fractions of haematological (orange) and solid tumours (blue). *P* values: binomial tests.

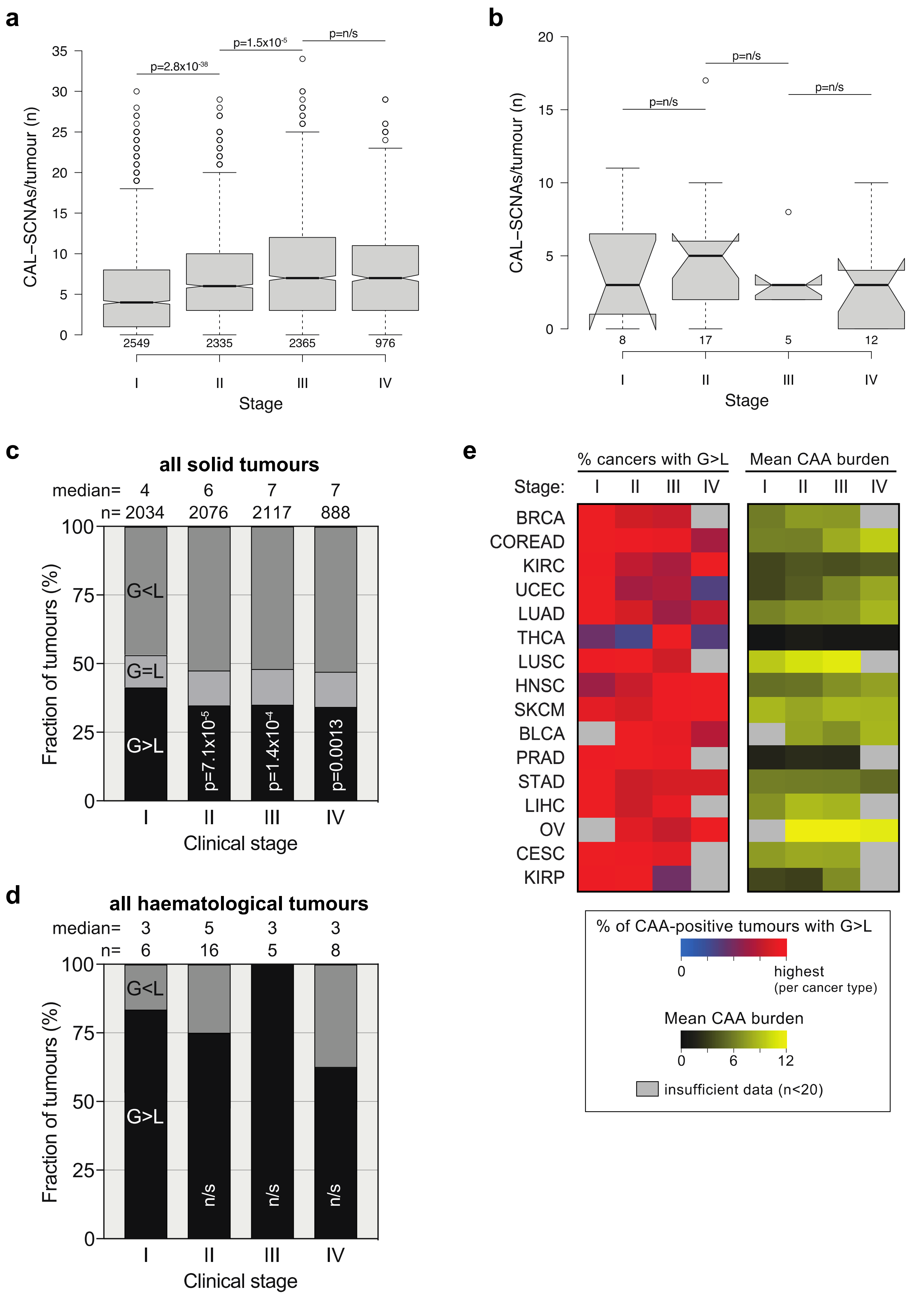
a
CAL–SCNAs/tumour (n)
p=2.8x10-38
p=1.5x10-5
p=n/s
2549
2335
2365
976
I
II
III
IV
Stage
b
CAL–SCNAs/tumour (n)
p=n/s
p=n/s
p=n/s
8
17
5
12
I
II
III
IV
Stage
c
all solid tumours
median= 4 6 7 7
n= 2034 2076 2117 888
Fraction of tumours (%)
G<L
G=L
G>L
p=7.1x10-5
p=1.4x10-4
p=0.0013
Clinical stage
d
all haematological tumours
median= 3 5 3 3
n= 6 16 5 8
Fraction of tumours (%)
G<L
G>L
n/s
n/s
n/s
Clinical stage
e
% cancers with G>L
Mean CAA burden
Stage: I II III IV
BRCA
COREAD
KIRC
UCEC
LUAD
THCA
LUSC
HNSC
SKCM
BLCA
PRAD
STAD
LIHC
OV
CESC
KIRP
% of CAA-positive tumours with G>L
0
highest
(per cancer type)
Mean CAA burden
0 6 12
insufficient data (n<20)

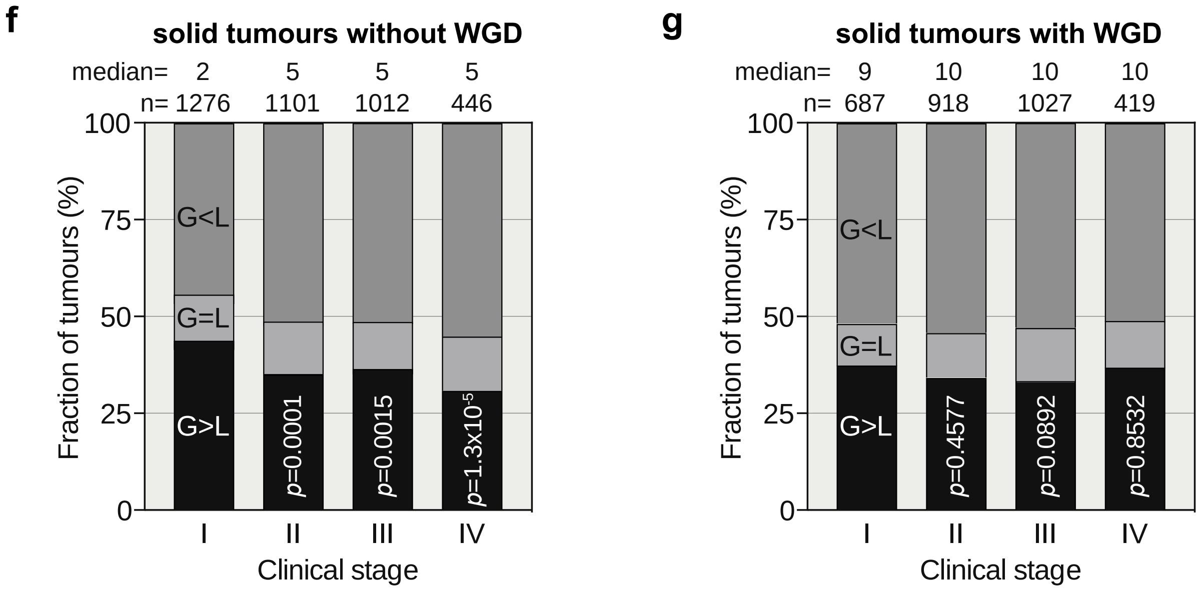

Supplementary Figure 4. CAA burden and intra-tumour chromosome arm gain:loss ratios in all samples and in samples with and without whole-genome doubling

(a,b) Tukey boxplots of CAA burden per clinical stage in solid cancers (a) and haematological cancers (b). Sample numbers are indicated below each box. *P* values: Mann-Whitney *U* tests. **(c, d)** Distributions of the fractions of CAA-positive tumours with more chromosome arm gains than losses (*G>L*), equal numbers of arm gains and losses (*G=L*) and more chromosome arm losses than gains (*G<L*) per solid tumour (c) or haematological cancer (d) according to clinical stage. Sample numbers and medians are shown on top. *P* values: Chi-square tests with respect to stage I cancers. **(e)** Heatmaps showing the fractions of indicated CAA-positive cancers with *G>L* per clinical stage (left) and the respective mean CAA burden (right). **(e, f)** Distributions of *G>L*, *G=L* and *G<L* fractions in solid tumours as in (a, b) but without (e) or with (f) whole-genome doubling (WGD).

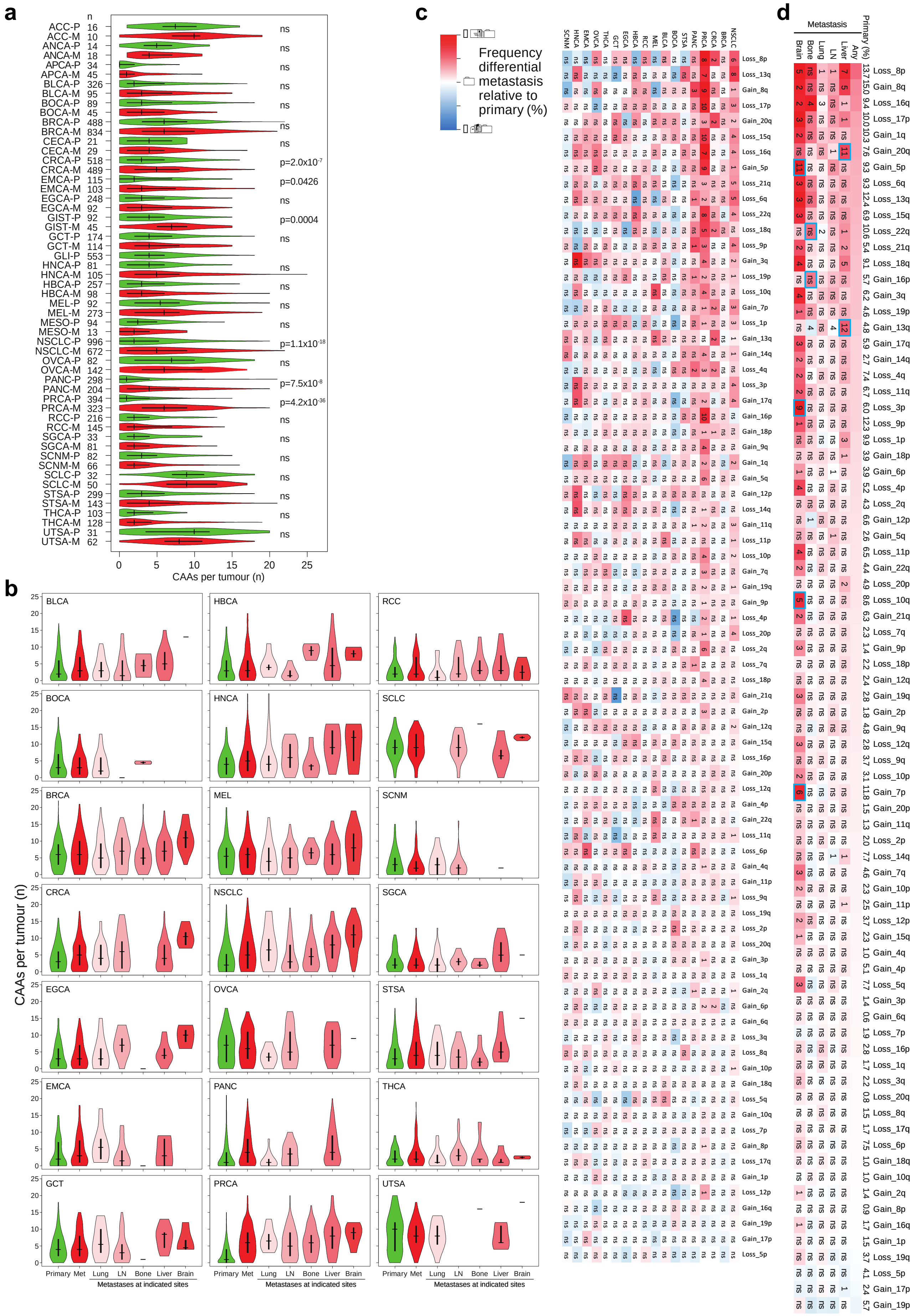
a
b
c
d
n
CAAs per tumour (n)
ns
p=2.0x10-7
p=0.0426
p=0.0004
p=1.1x10-18
p=7.5x10-8
p=4.2x10-36
BLCA
HBCA
RCC
BOCA
HNCA
SCLC
BRCA
MEL
SCNM
CRCA
NSCLC
SGCA
EGCA
OVCA
STSA
EMCA
PANC
THCA
GCT
PRCA
UTSA
Primary Met Lung LN Bone Liver Brain
Metastases at indicated sites
Frequency differential metastasis relative to primary (%)
Metastasis
Brain Bone Lung LN Liver Any
Primary (%)

Supplementary Figure 5 (previous page). CAA burden in primary and metastatic cancers
(a) Violin plots comparing the CAA burden in cancer type-matched primary (P) and metastatic (M) tumour samples. Median and interquartile ranges are shown within each violin. *P* values: Mann-Whitney *U* tests. **(b)** CAA burden as in (a) but specifying metastatic sites. **(c)** Heatmap of the percentage frequency increase (red) or decrease (blue) per CAA in metastatic disease relative to the CAA frequencies in respective primary cancers. Text in each tile refers to significance levels of FDR-adjusted *q* values: ns, not significant ($q > 0.05$); 1, $q < 0.05$; 2, $q < 10^{-2}$; 3, $q < 10^{-3}$, etc. **(d)** Heatmap as in (c) but for combined solid cancers and relative to specific metastatic sites.

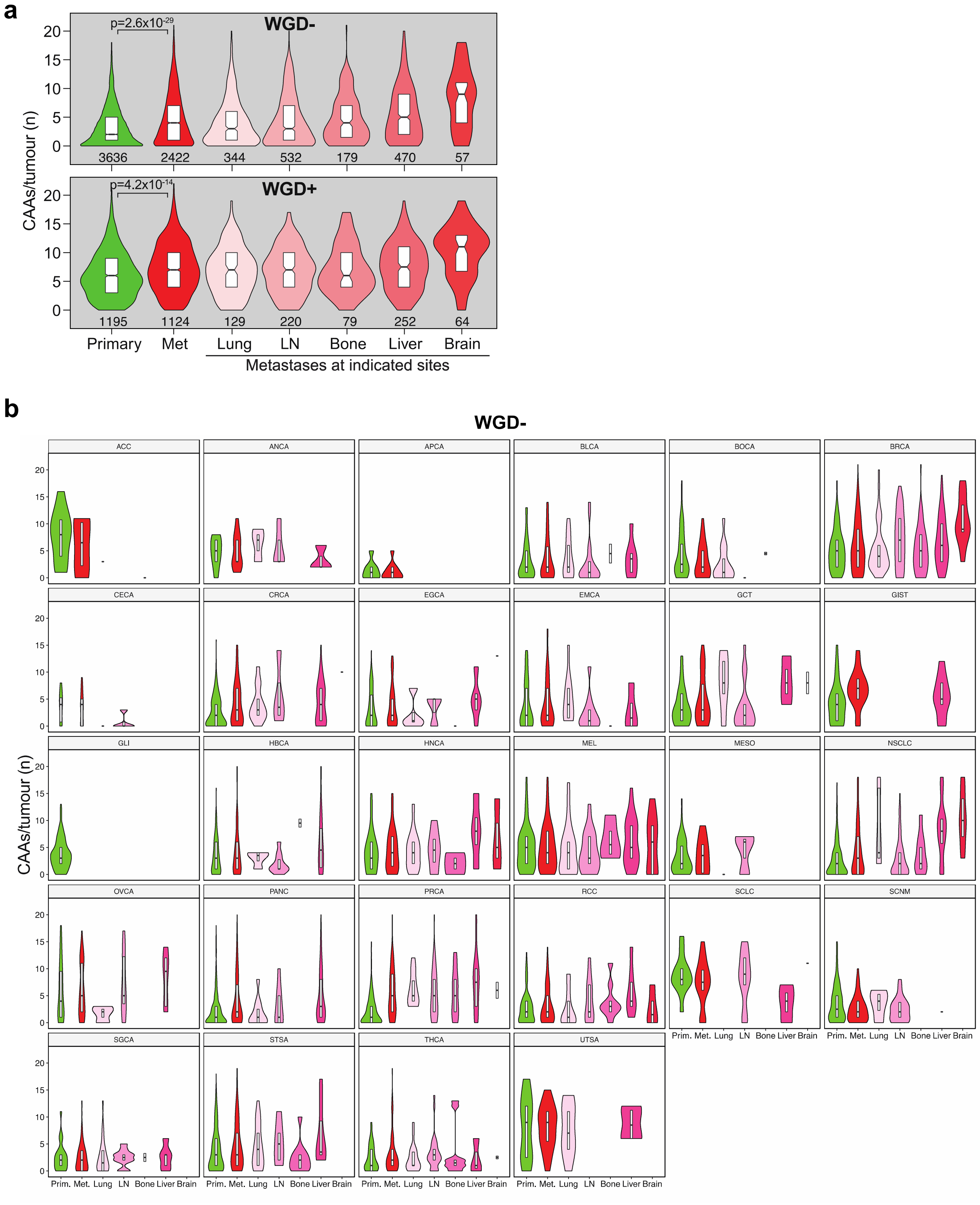
a
WGD-
p=2.6x10-29
20
15
10
5
0
3636
2422
344
532
179
470
57
WGD+
p=4.2x10-14
1195
1124
129
220
79
252
64
CAAs/tumour (n)
Primary
Met
Lung
LN
Bone
Liver
Brain
Metastases at indicated sites
b
WGD-
ACC
ANCA
APCA
BLCA
BOCA
BRCA
CECA
CRCA
EGCA
EMCA
GCT
GIST
GLI
HBCA
HNCA
MEL
MESO
NSCLC
OVCA
PANC
PRCA
RCC
SCLC
SCNM
SGCA
STSA
THCA
UTSA
Prim. Met. Lung LN Bone Liver Brain

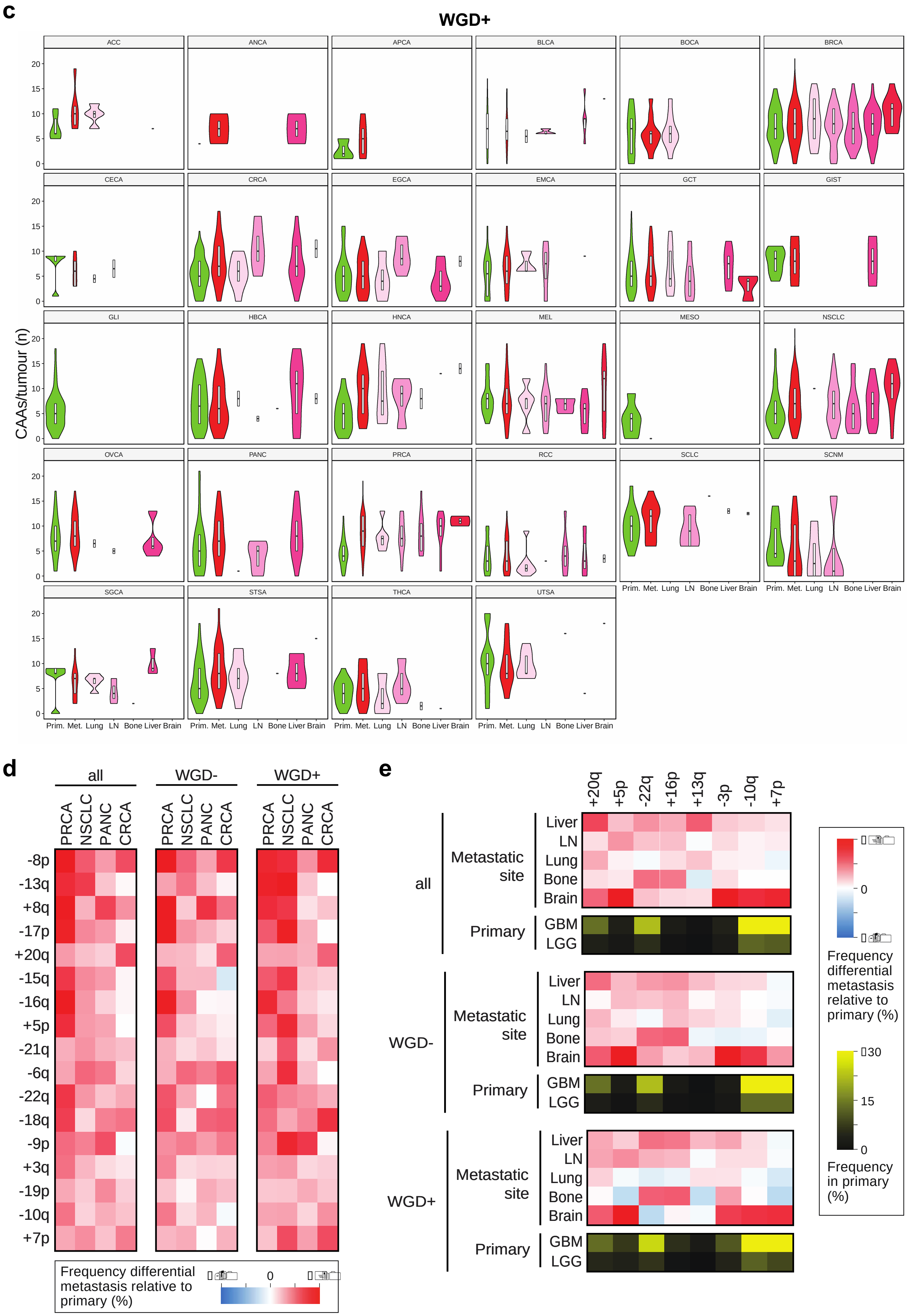
c
WGD+
ACC
ANCA
APCA
BLCA
BOCA
BRCA
CECA
CRCA
EGCA
EMCA
GCT
GIST
GLI
HBCA
HNCA
MEL
MESO
NSCLC
OVCA
PANC
PRCA
RCC
SCLC
SCNM
SGCA
STSA
THCA
UTSA
CAAs/tumour (n)
0
5
10
15
20
Prim. Met. Lung LN Bone Liver Brain
d
all
WGD-
WGD+
PRCA NSCLC PANC CRCA
-8p
-13q
+8q
-17p
+20q
-15q
-16q
+5p
-21q
-6q
-22q
-18q
-9p
+3q
-19p
-10q
+7p
Frequency differential metastasis relative to primary (%)
0
e
+20q
+5p
-22q
+16p
+13q
-3p
-10q
+7p
all
WGD-
WGD+
Metastatic site
Primary
Liver
LN
Lung
Bone
Brain
GBM
LGG
0
Frequency differential metastasis relative to primary (%)
30
15
0
Frequency in primary (%)

Supplementary Figure 6 (previous pages). CAA burden in primary and metastatic cancers with and without whole-genome doubling

(a-e) Violin plots as in Fig. 2a (a) and Suppl. Fig. S5b (b,c), respectively, and heatmaps as in Fig. 2b (d) and Fig. 2c (e), respectively, but samples are separated in whole-genome doubling-negative (WGD-) and -positive (WGD+) groups, as indicated.

Supplementary Figure 7. Stochastic tumour evolution modelling

(a) A multi-level tree, $G = (V, E)$, was created in which each node represents a 'CAA karyotype', a one-dimensional array A with 78 elements, one for each possible CAA, which can be absent, $'0'$, or present, $'1'$ (a). **(b)** A hypothetical example of such tree, used here to illustrate how transition probabilities were computed (see also Methods). All nodes with k CAAs per karyotype belong to level k. In this example, Level 1 has two nodes: one with $-2q$ (appears 2 times in this dataset) and one with $+16p$ (appears 10 times in this dataset). The transition $\{-2q \rightarrow +4p\}$ appears four times in this tree. Its unnormalized weight $W(-2q \rightarrow +4p) = 8 + 18 + 7 + 9 = 42$. Similarly, $W(-2q \rightarrow +16p) = 20$. $W(-2q \rightarrow +22p) = 12 + 6 + 7 = 25$ and $W(-2q \rightarrow +3q) = 4 + 9 = 13$. Therefore, the estimation of the probability of the transition $\{-2q \rightarrow +4p\}$ is $42/(42 + 20 + 25 + 13) = 0.42$.

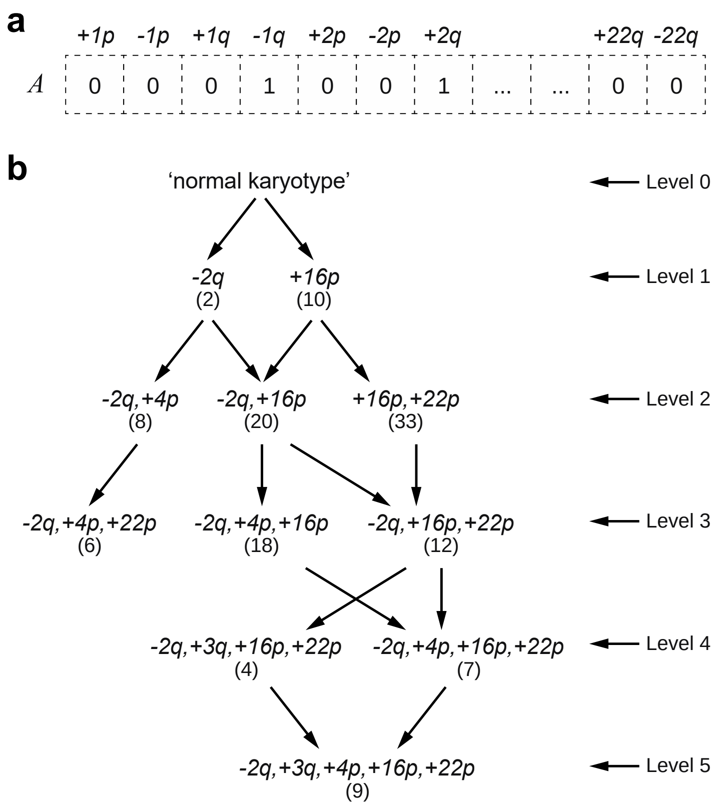

Supplementary Figure 8. Fractions of patients with significant good and poor survival-predicting CAAs

Meta-analysis of the numbers of significant good and poor survival-predicting CAAs from pan-cancer multivariate Cox proportional hazard modelling. Fractions of patients affected by each significant association with good or poor disease-free or overall survival are shown. Black lines indicate means with standard errors of the means. P values: Mann-Whitney U tests.

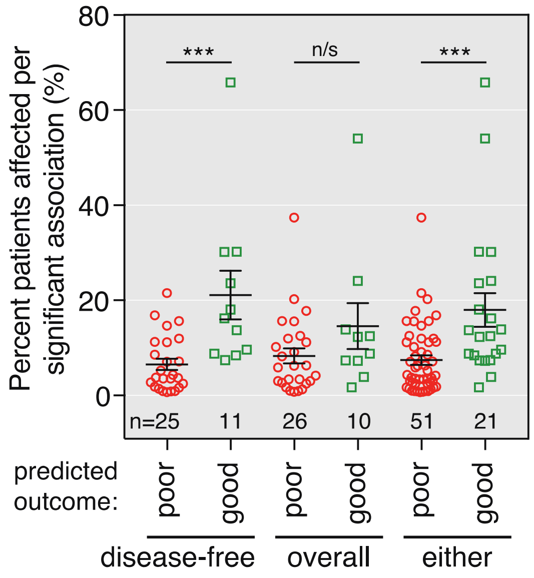

ACC (n=90; max p−value=0.04732)

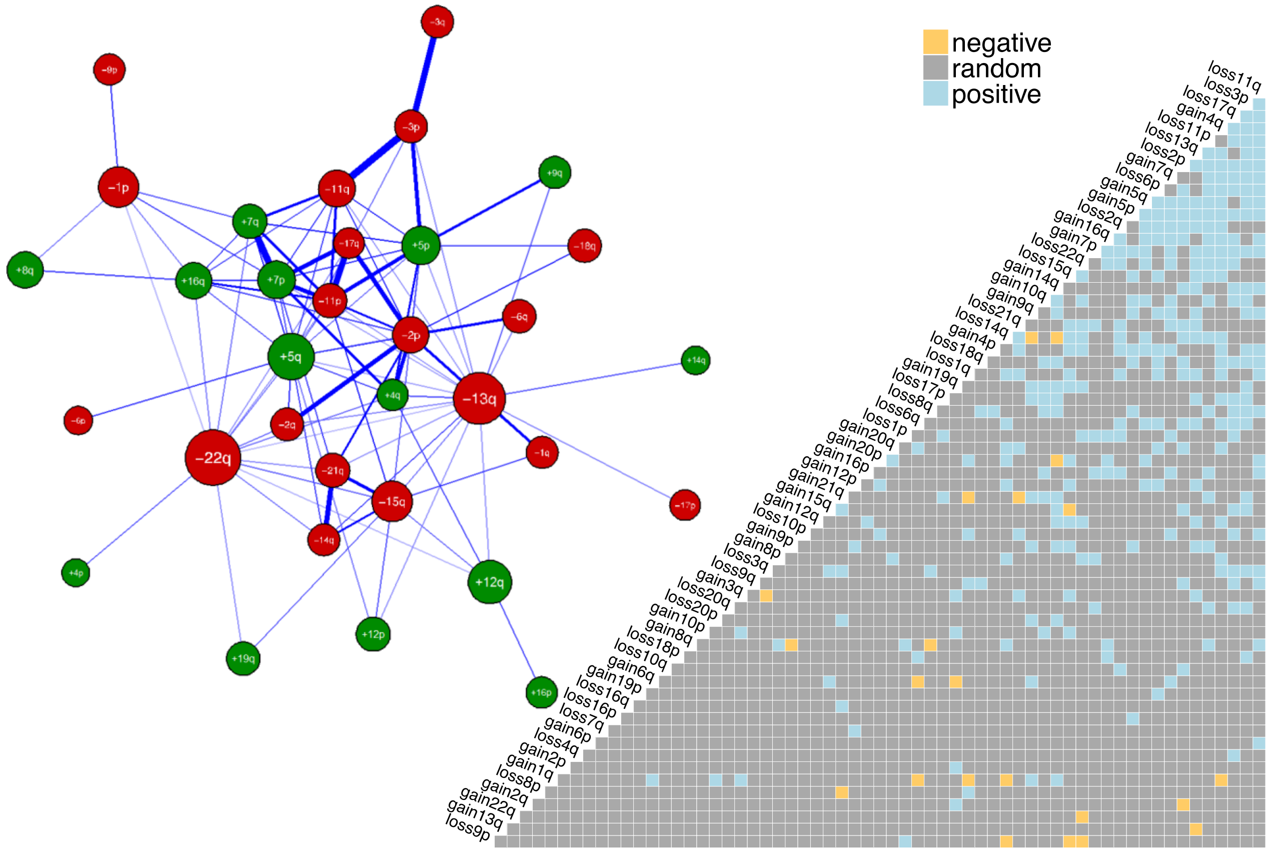

BLCA (n=411; max p−value=0.0467)

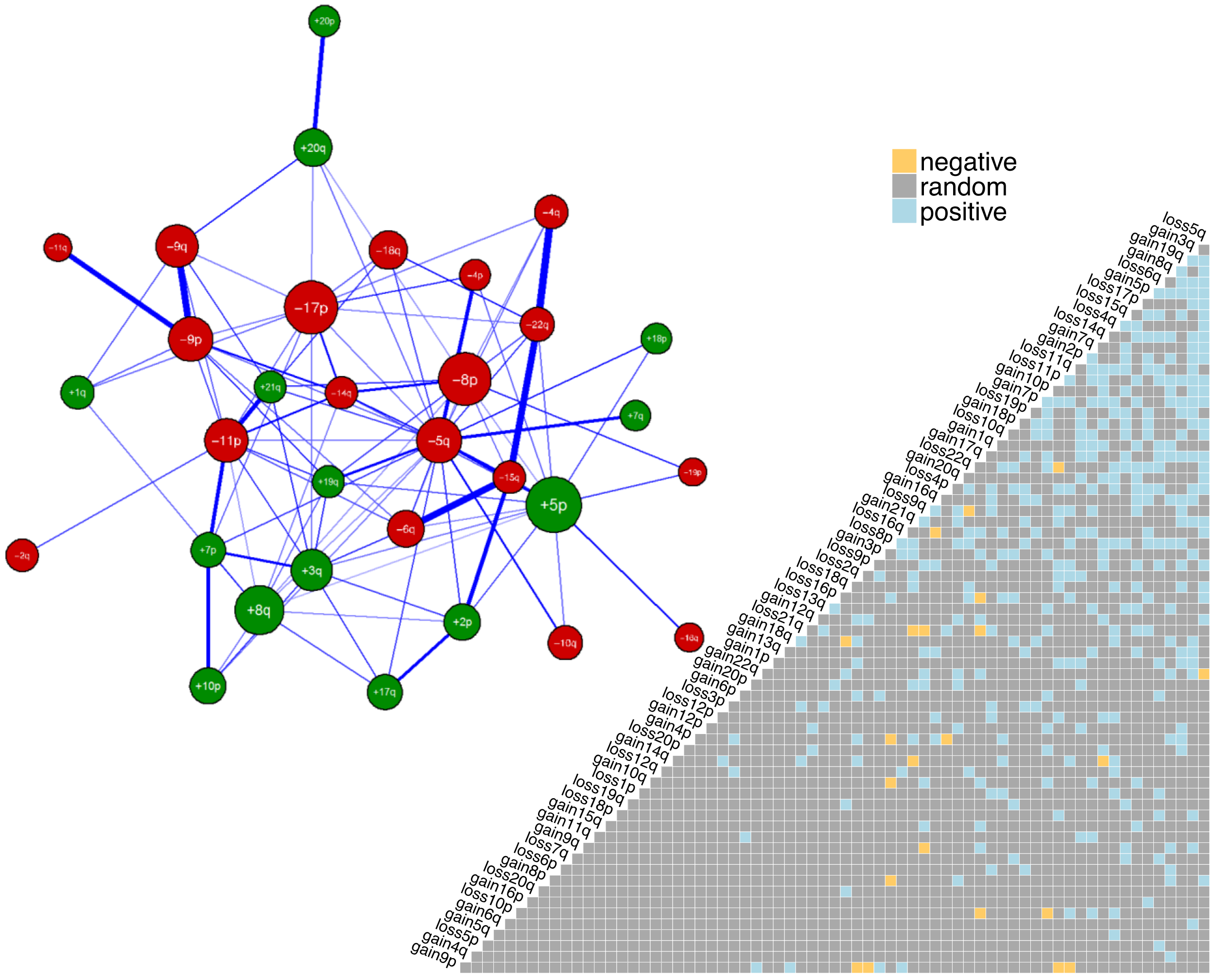

BRCA (n=1094; max p−value=0.04701)

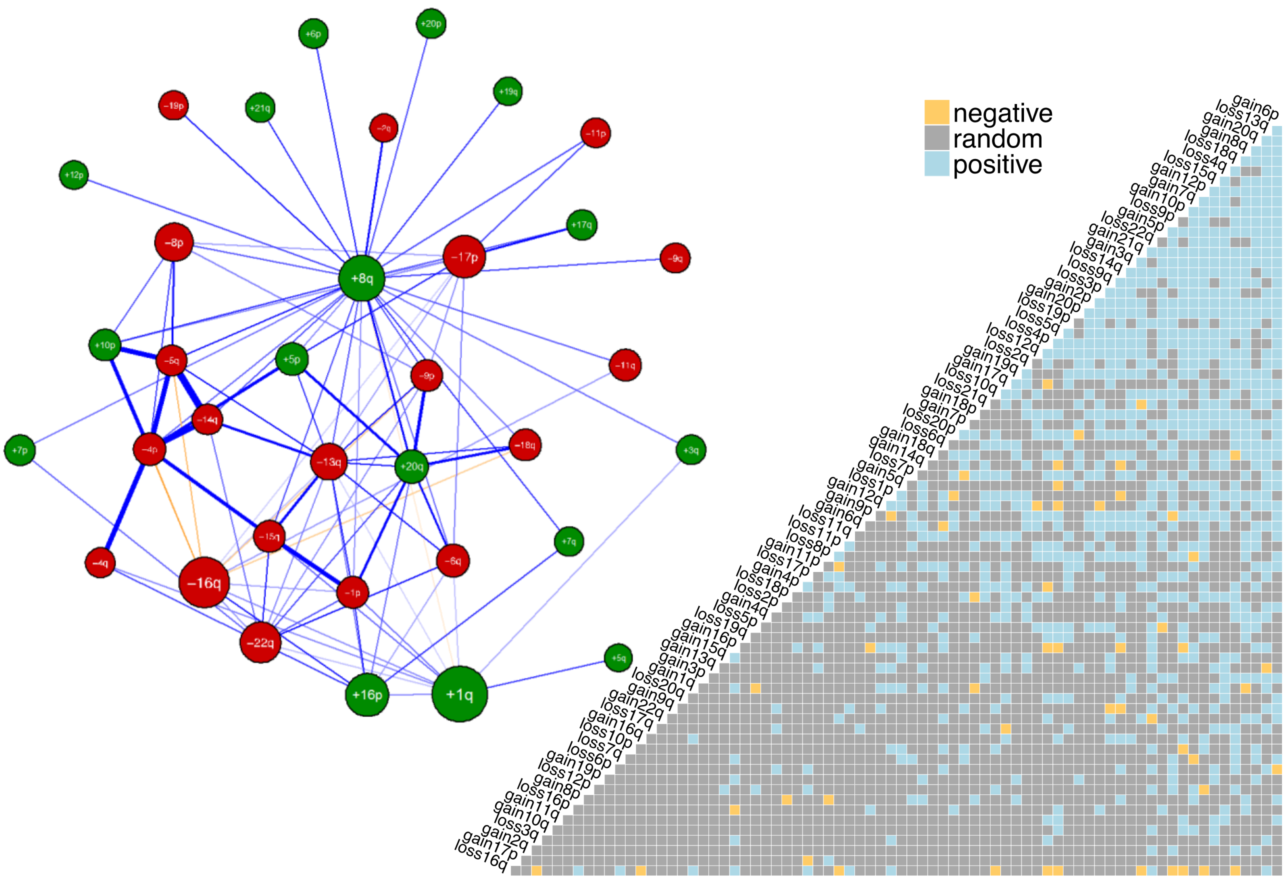

CESC (n=297; max p−value=0.04803)

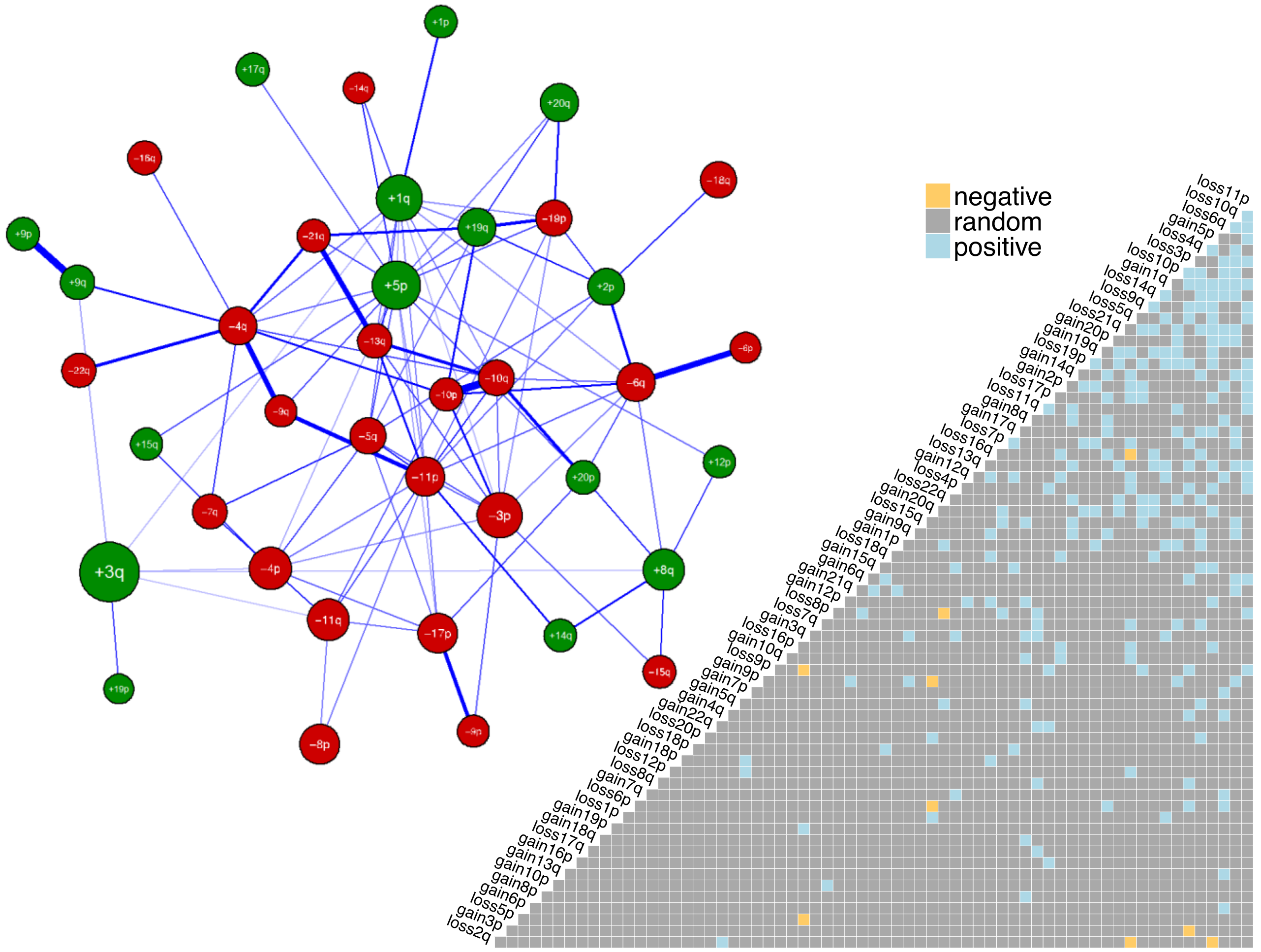

CHOL (n=36; max p−value=0.04698)

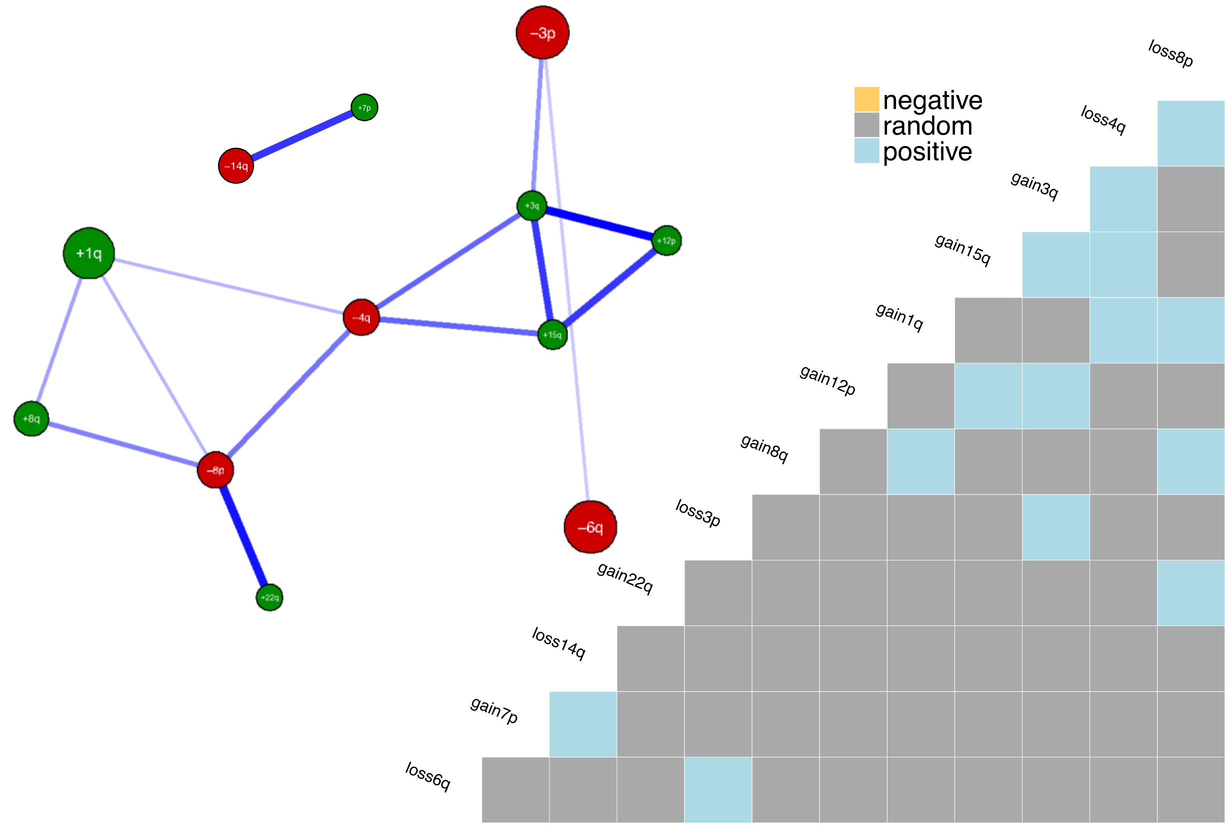

COADREAD (n=631; max p−value=0.04995)

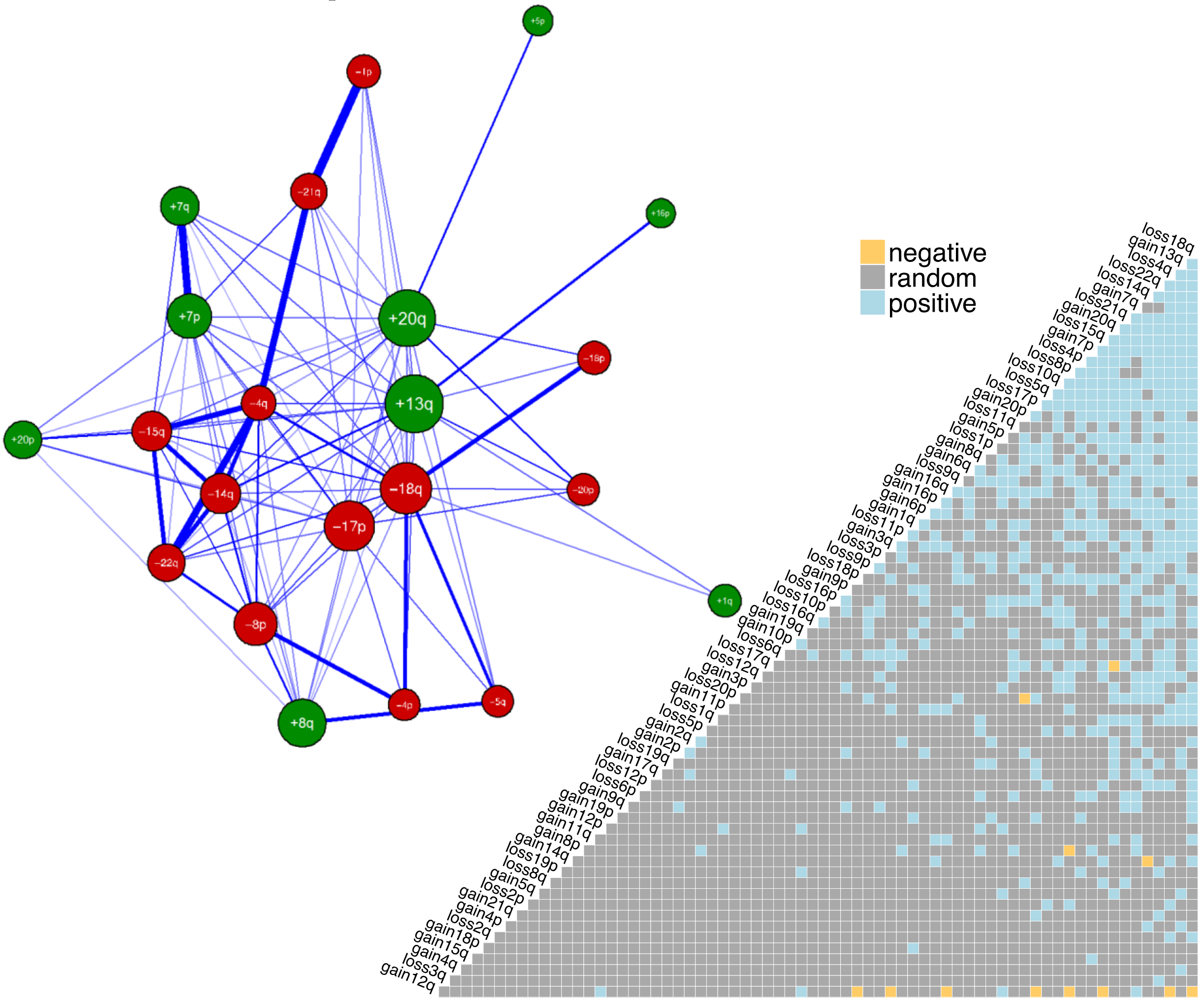

DLBC (n=48; max p−value=0.04325)

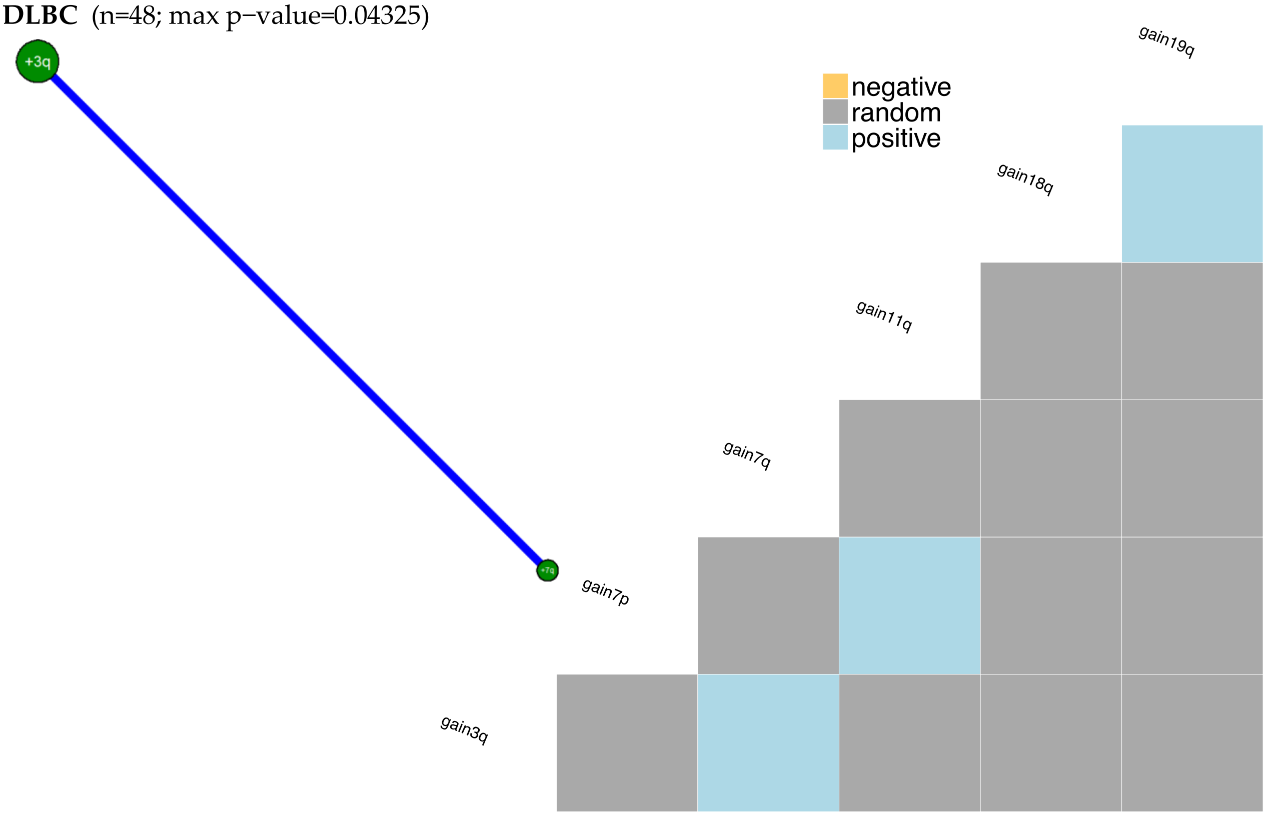

ESCA (n=185; max p−value=0.04822)

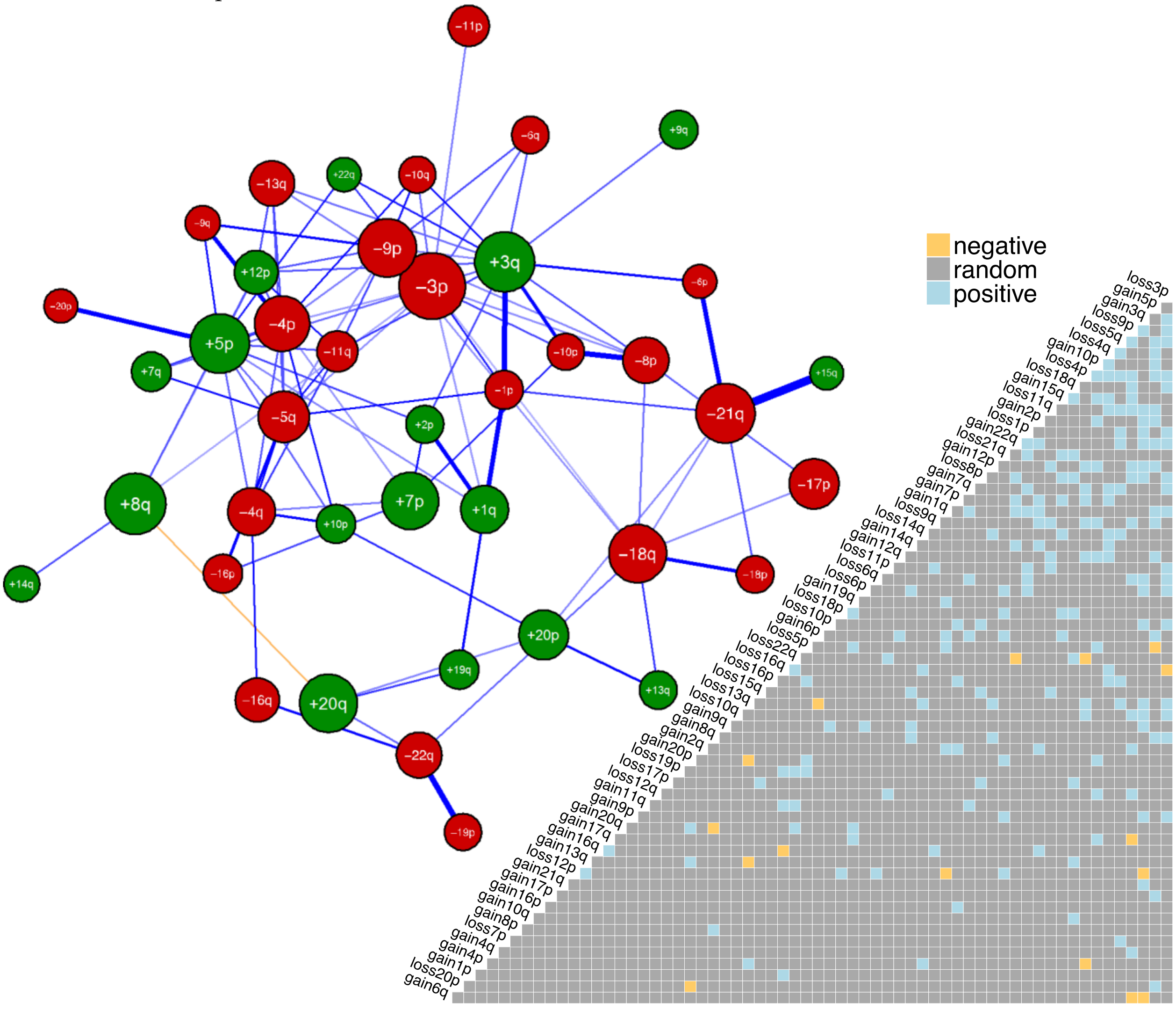

GBM (n=590; max p−value=0.04928)

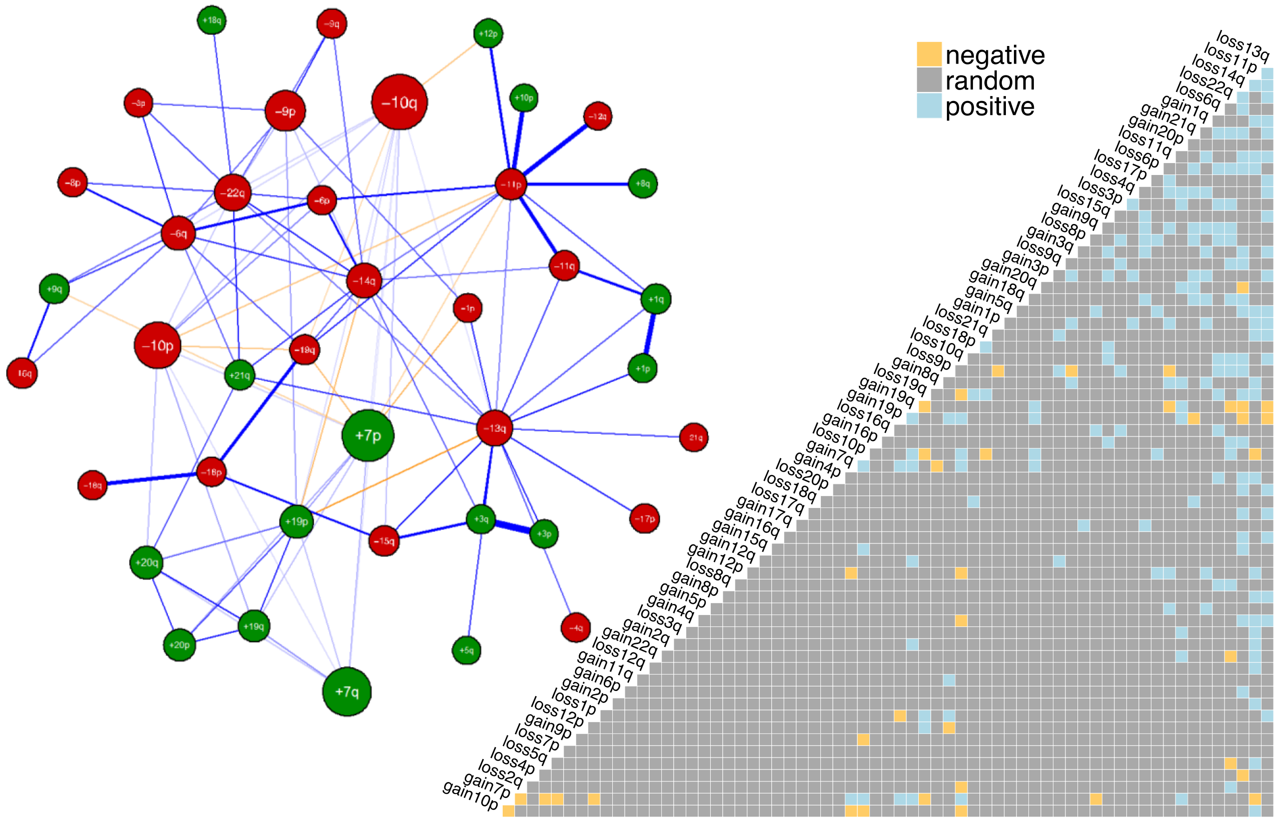

HNSC (n=524; max p−value=0.04867)

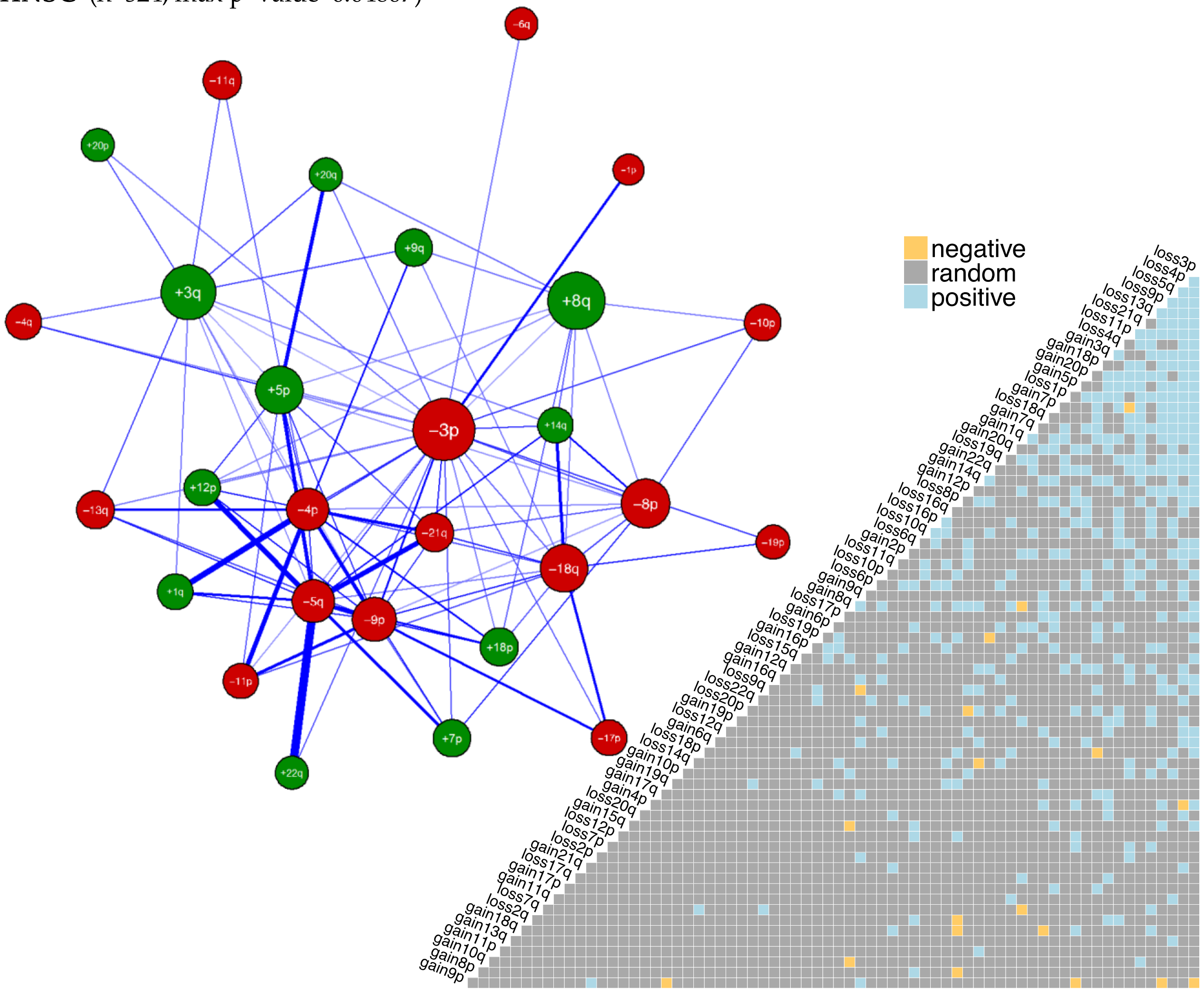

KIRC (n=532; max p−value=0.04807)

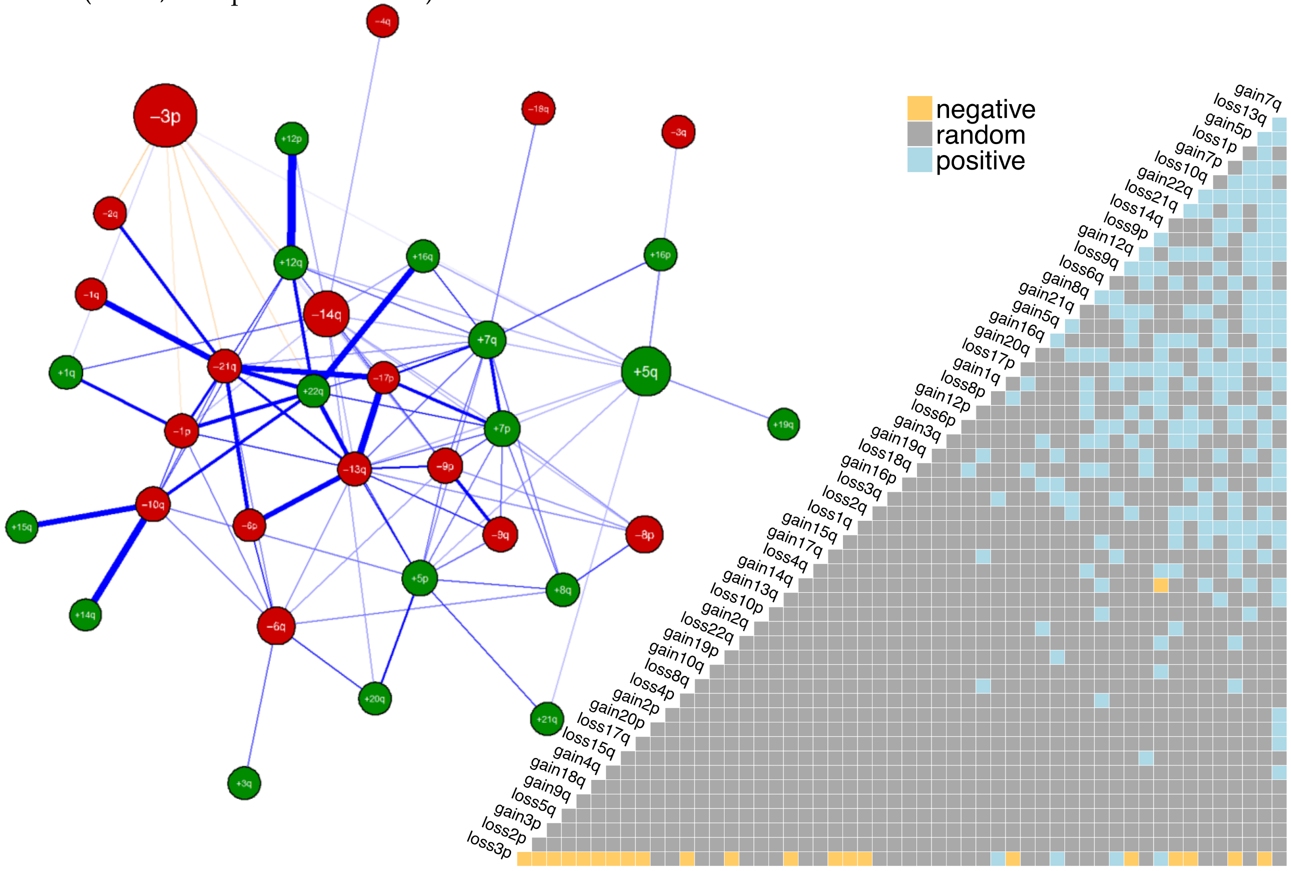

KIRP (n=288; max p−value=0.04801)

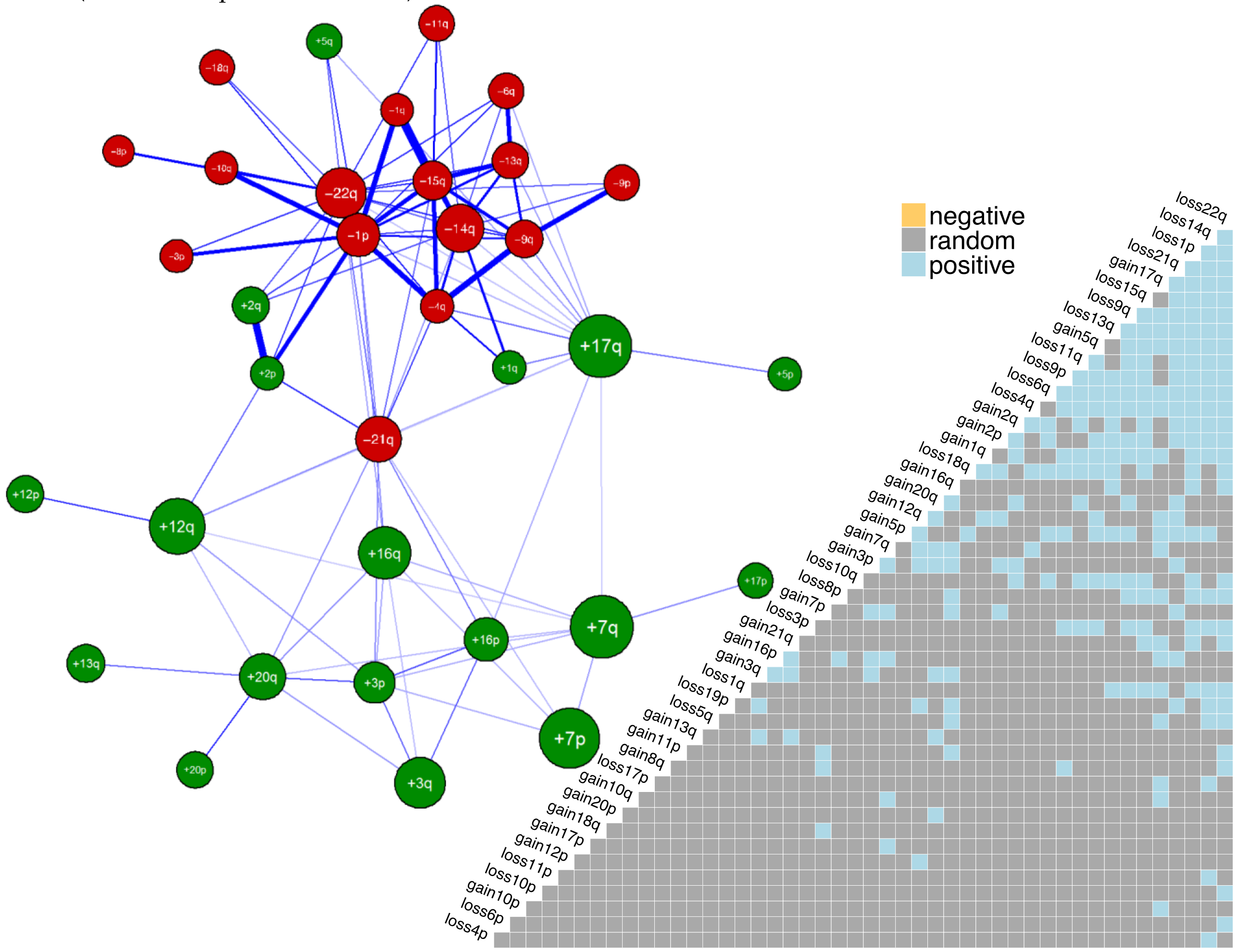

LAML (n=191; max p−value=0.02095)

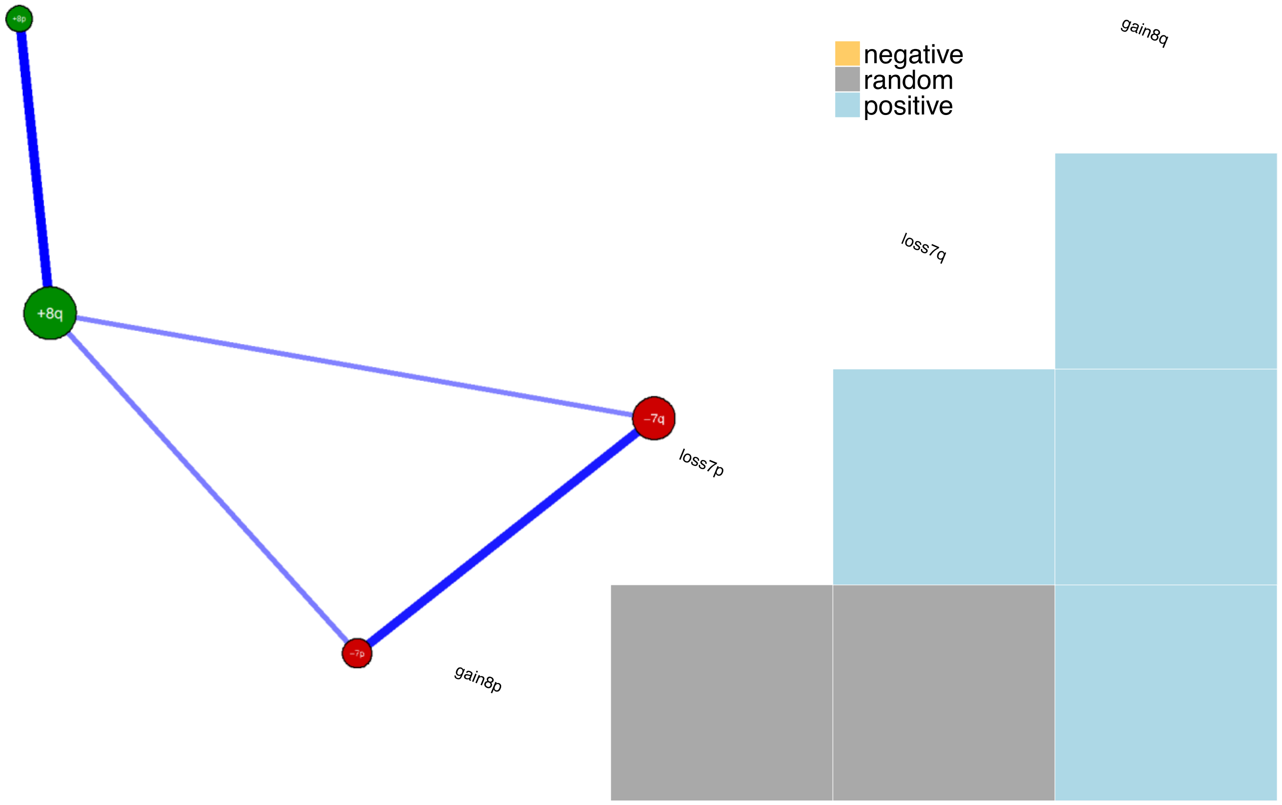

LGG (n=527; max p−value=0.04818)

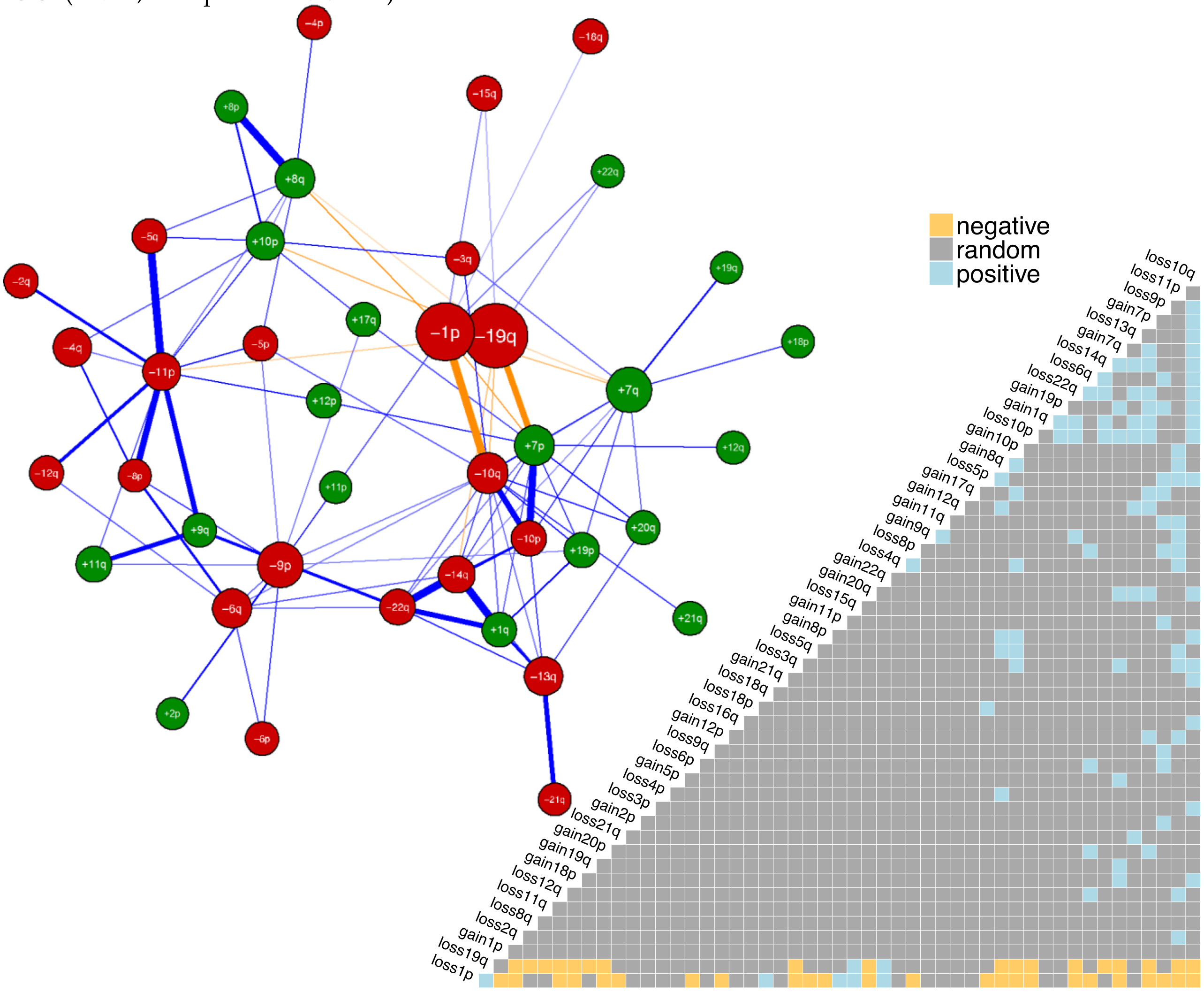

LIHC (n=372; max p−value=0.0485)

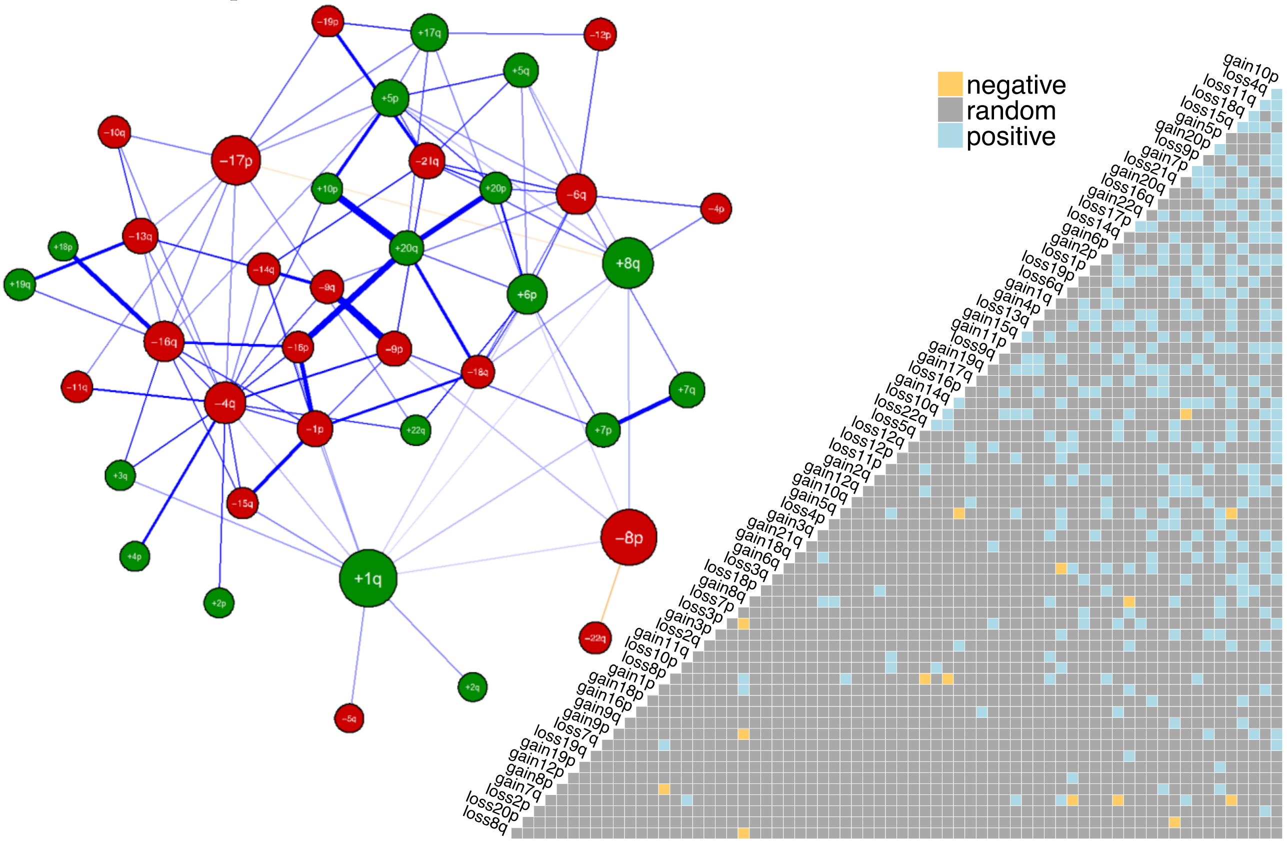

LUAD (n=530; max p−value=0.04847)

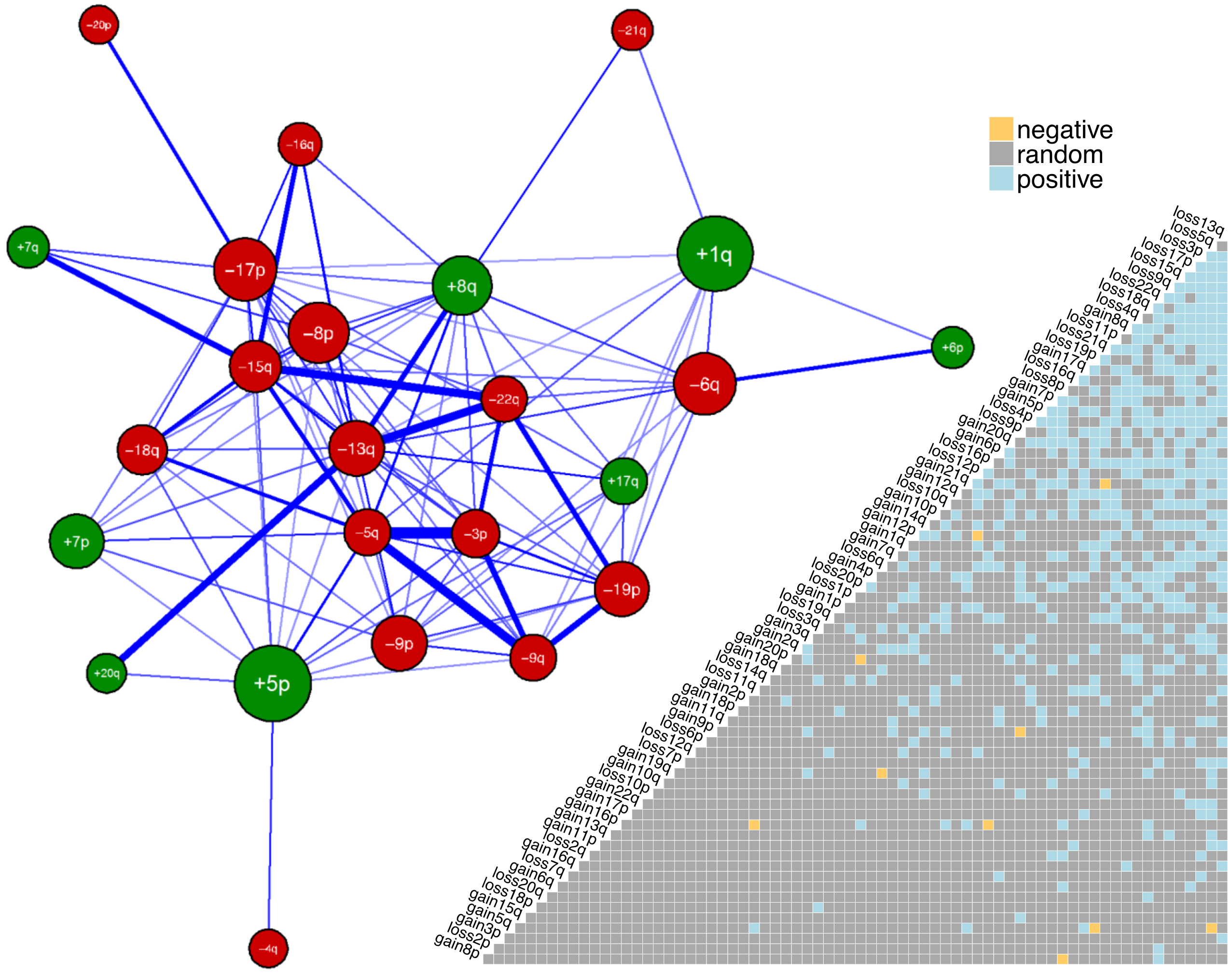

LUSC (n=501; max p−value=0.04812)

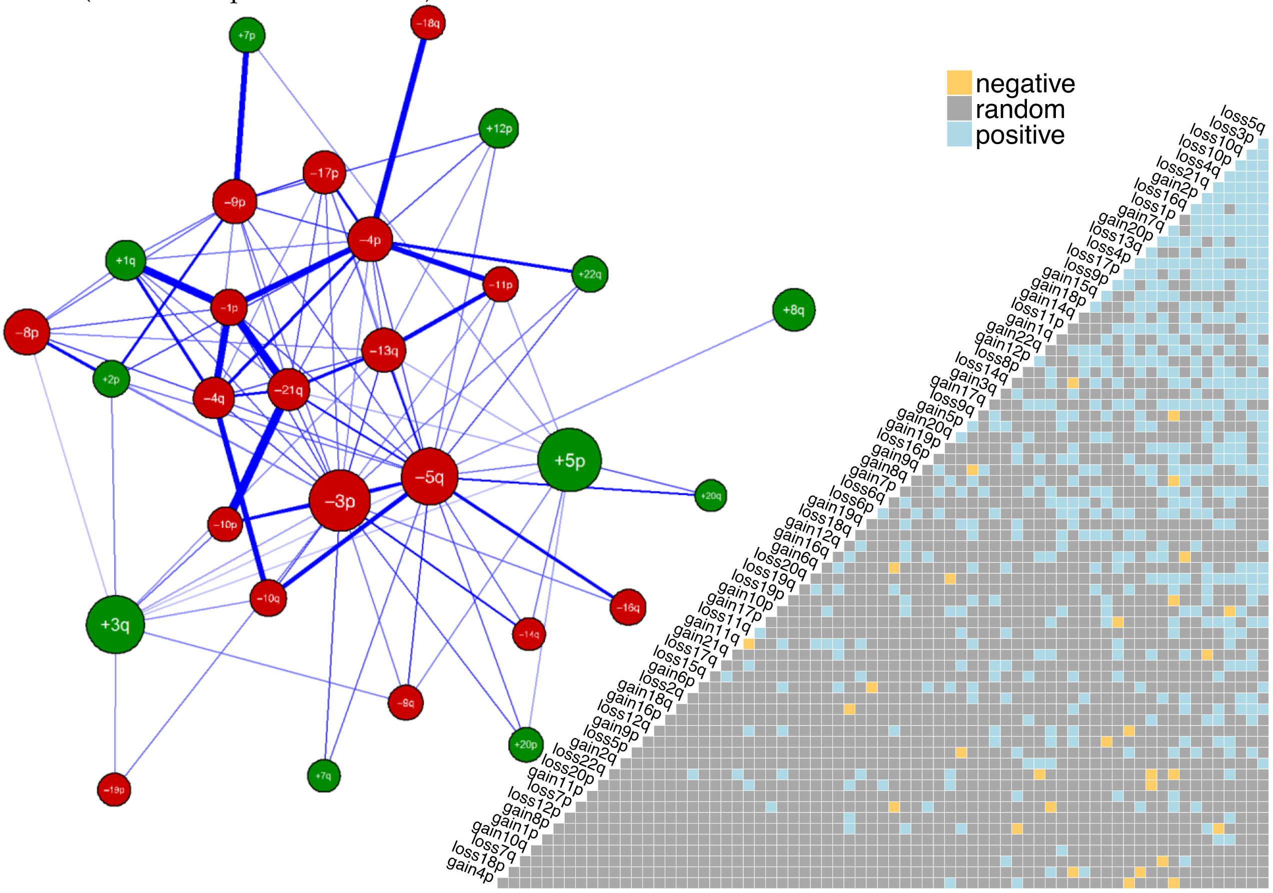

MESO (n=87; max p−value=0.04826)

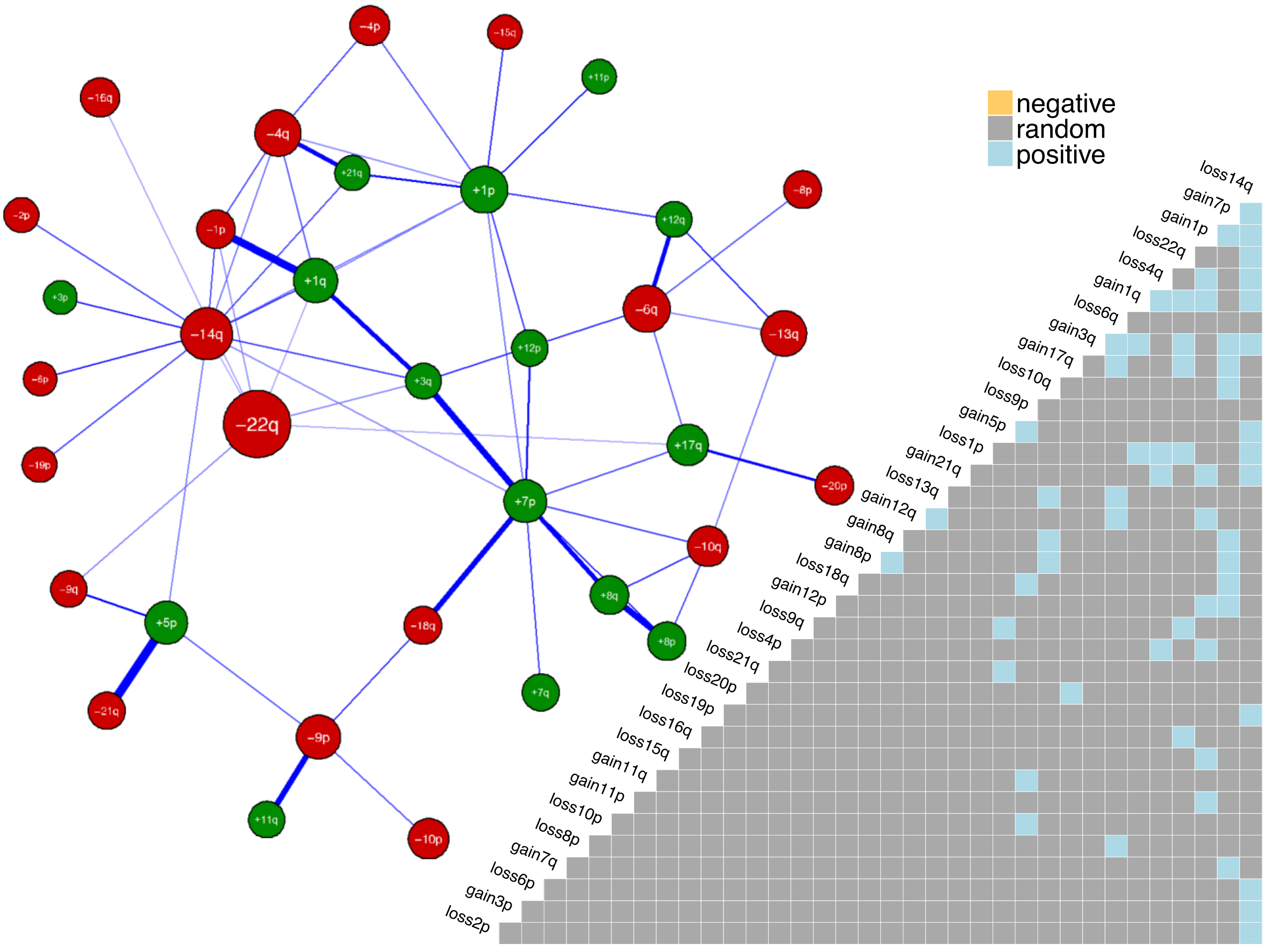

OV (n=597; max p−value=0.0495)

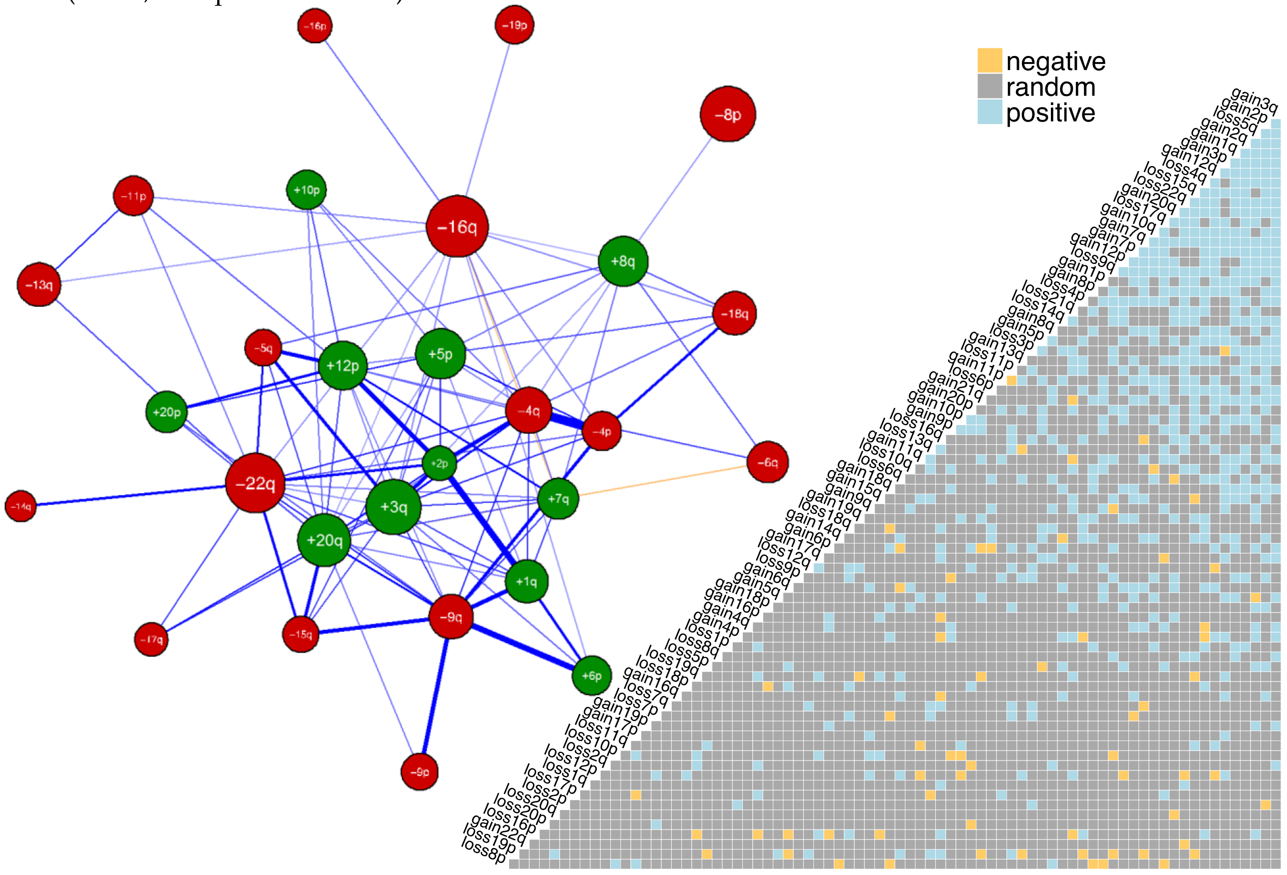

PAAD (n=185; max p−value=0.04645)

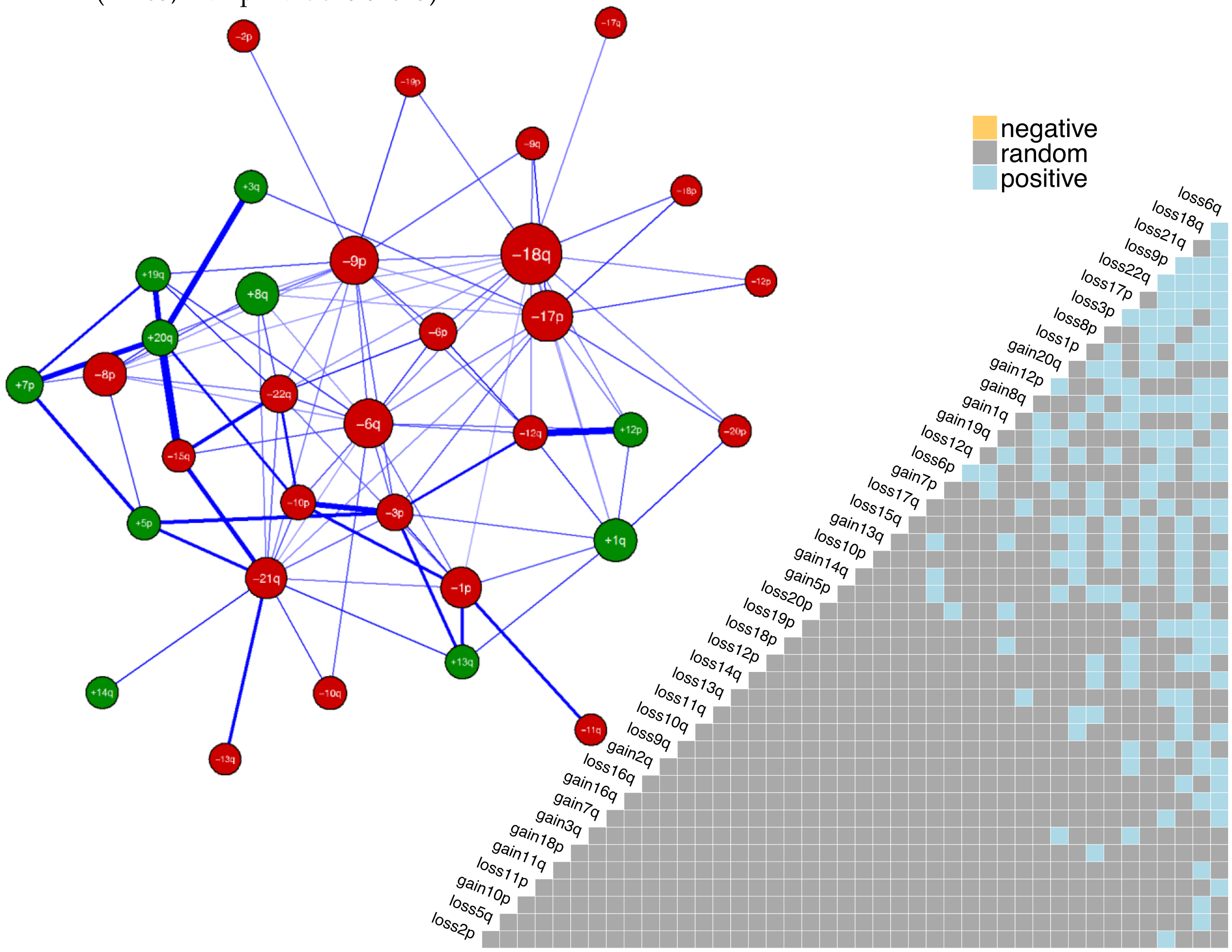

PCPG (n=166; max p−value=0.04689)

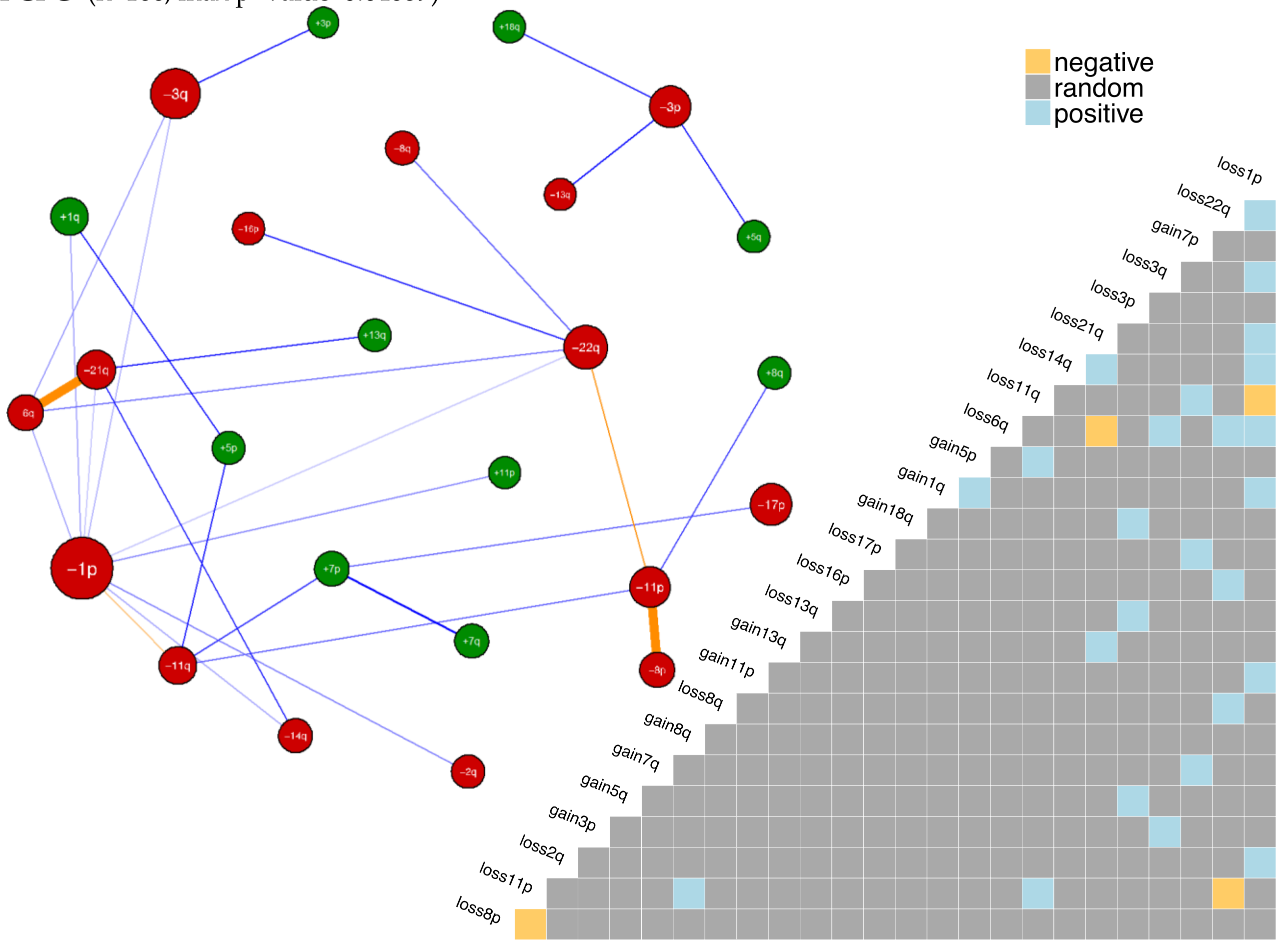

PRAD (n=499; max p−value=0.04944)

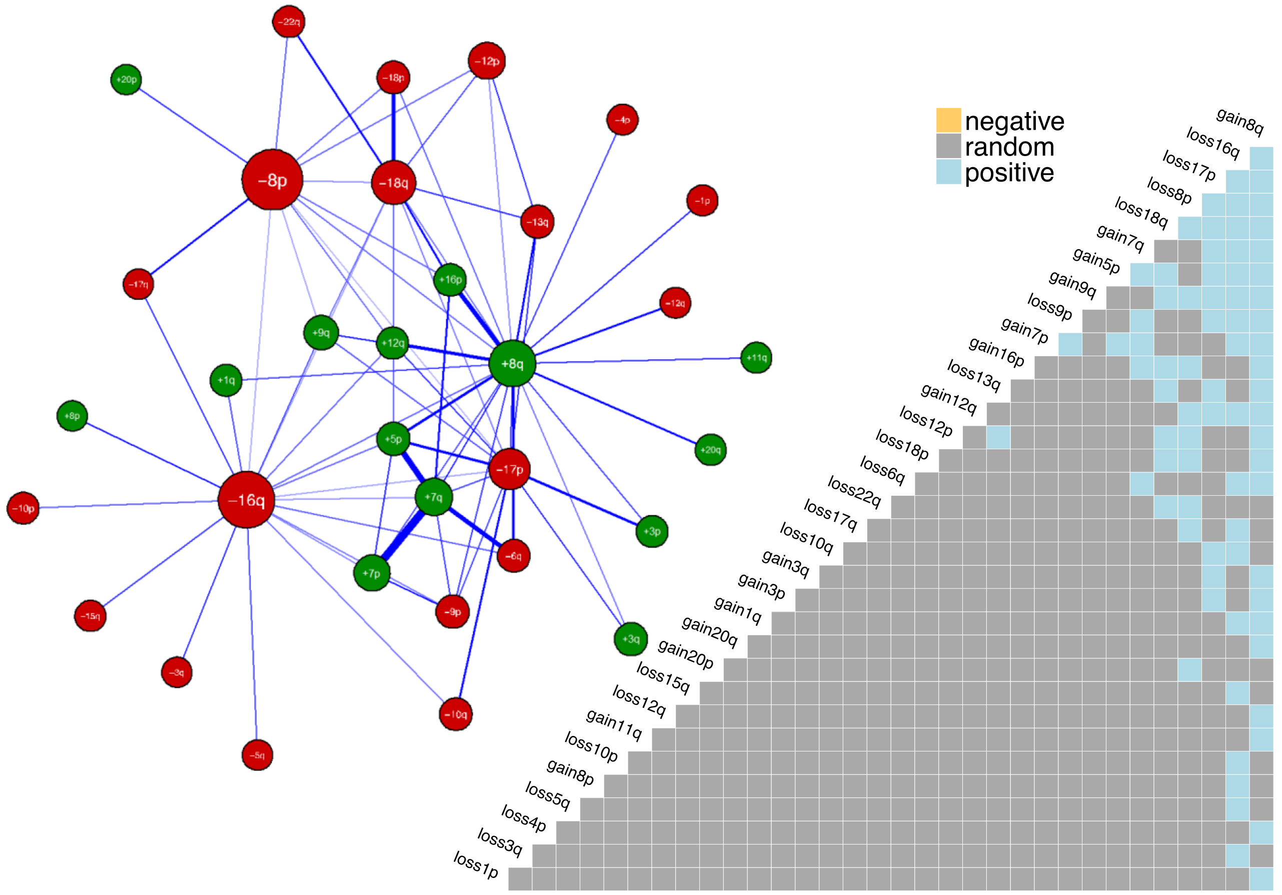

SARC (n=261; max p−value=0.04836)

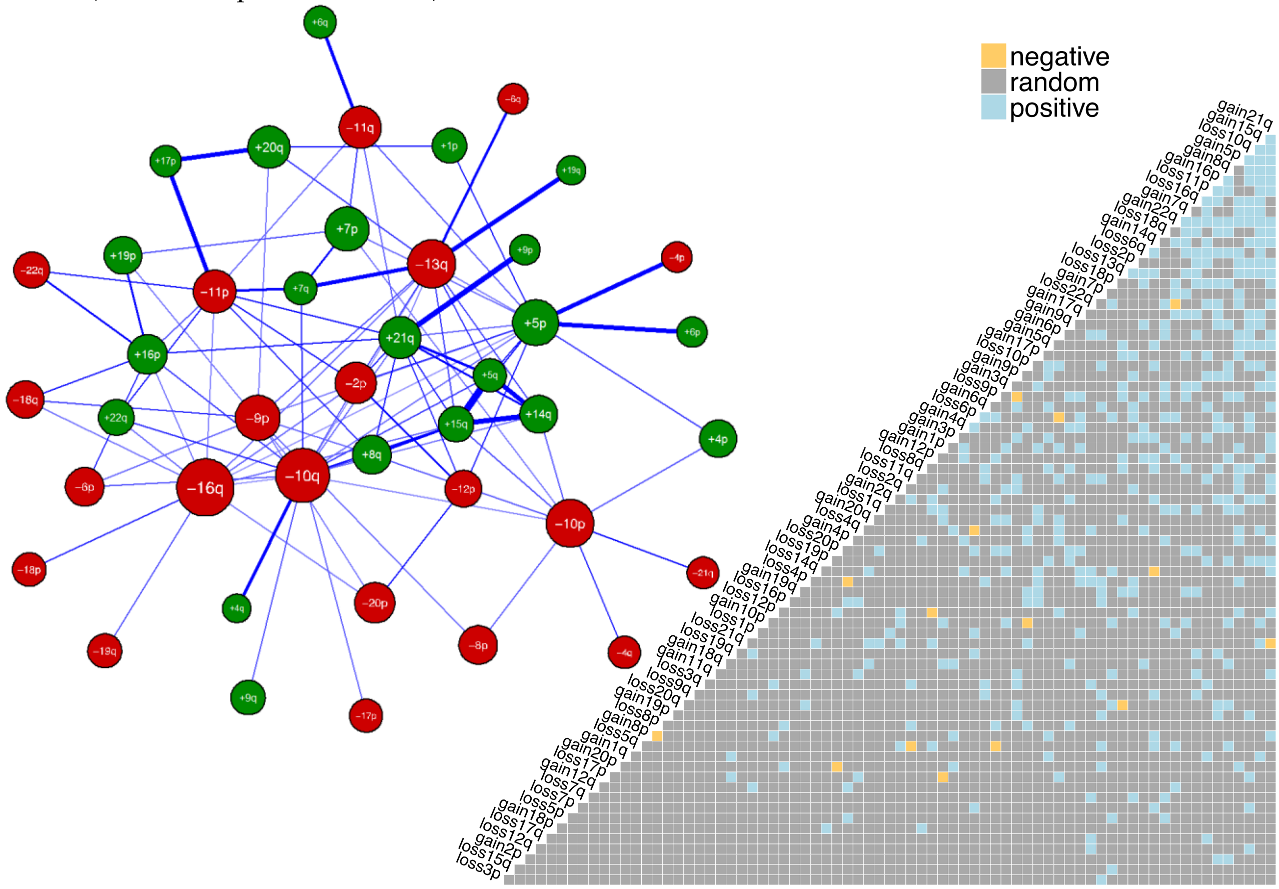

SKCM (n=471; max p−value=0.0482)

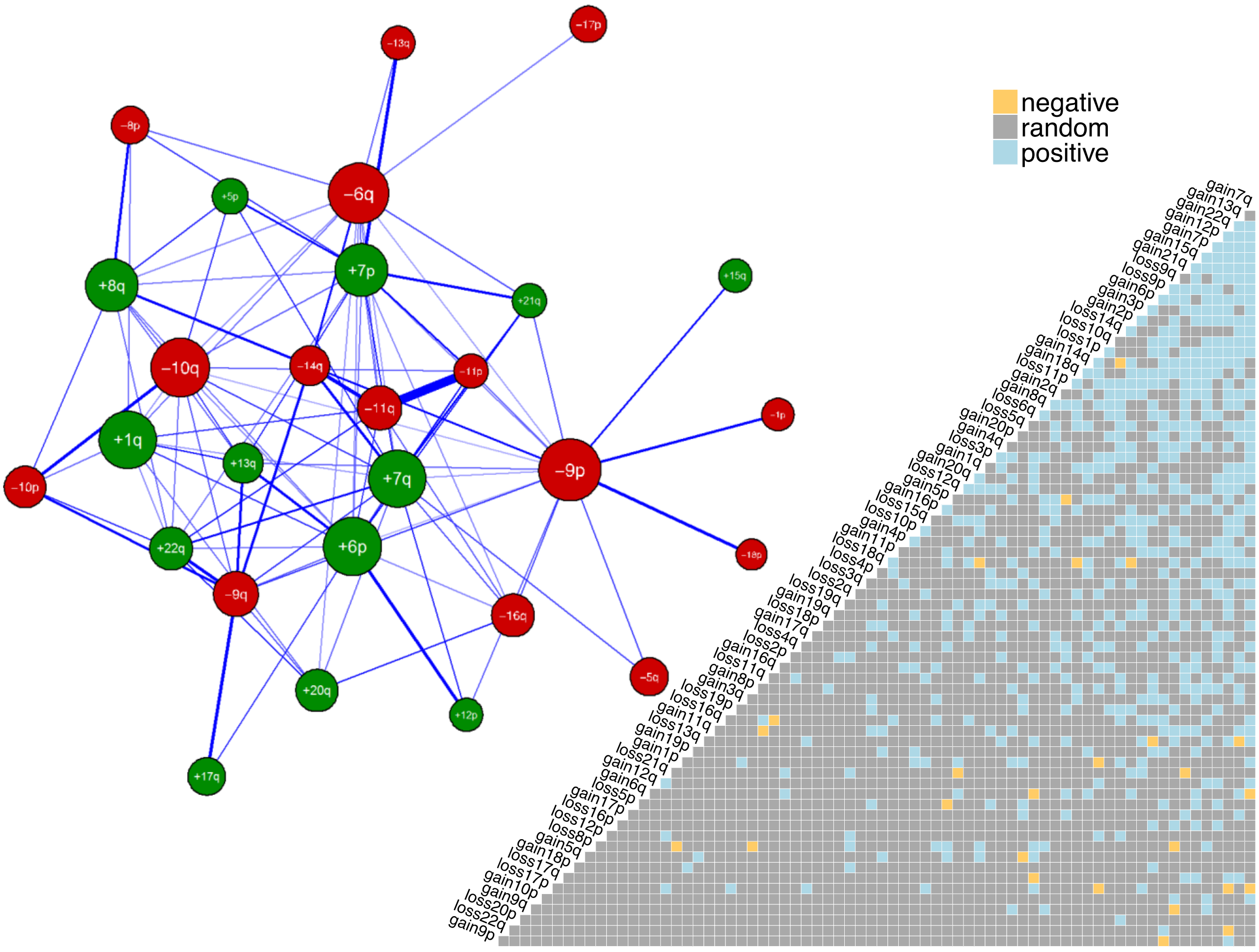

STAD (n=441; max p−value=0.03251)

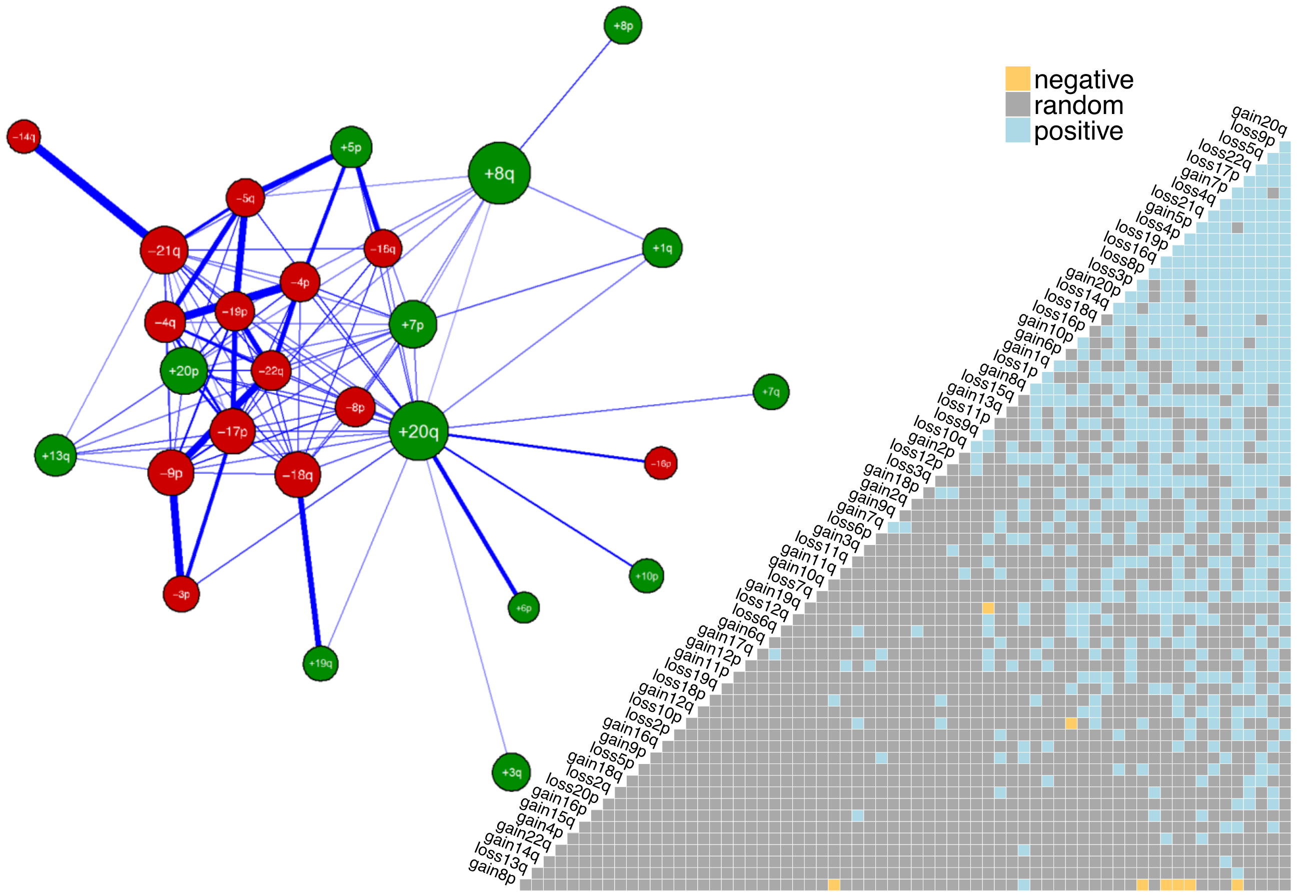

TGCT (n=156; max p−value=0.04683)

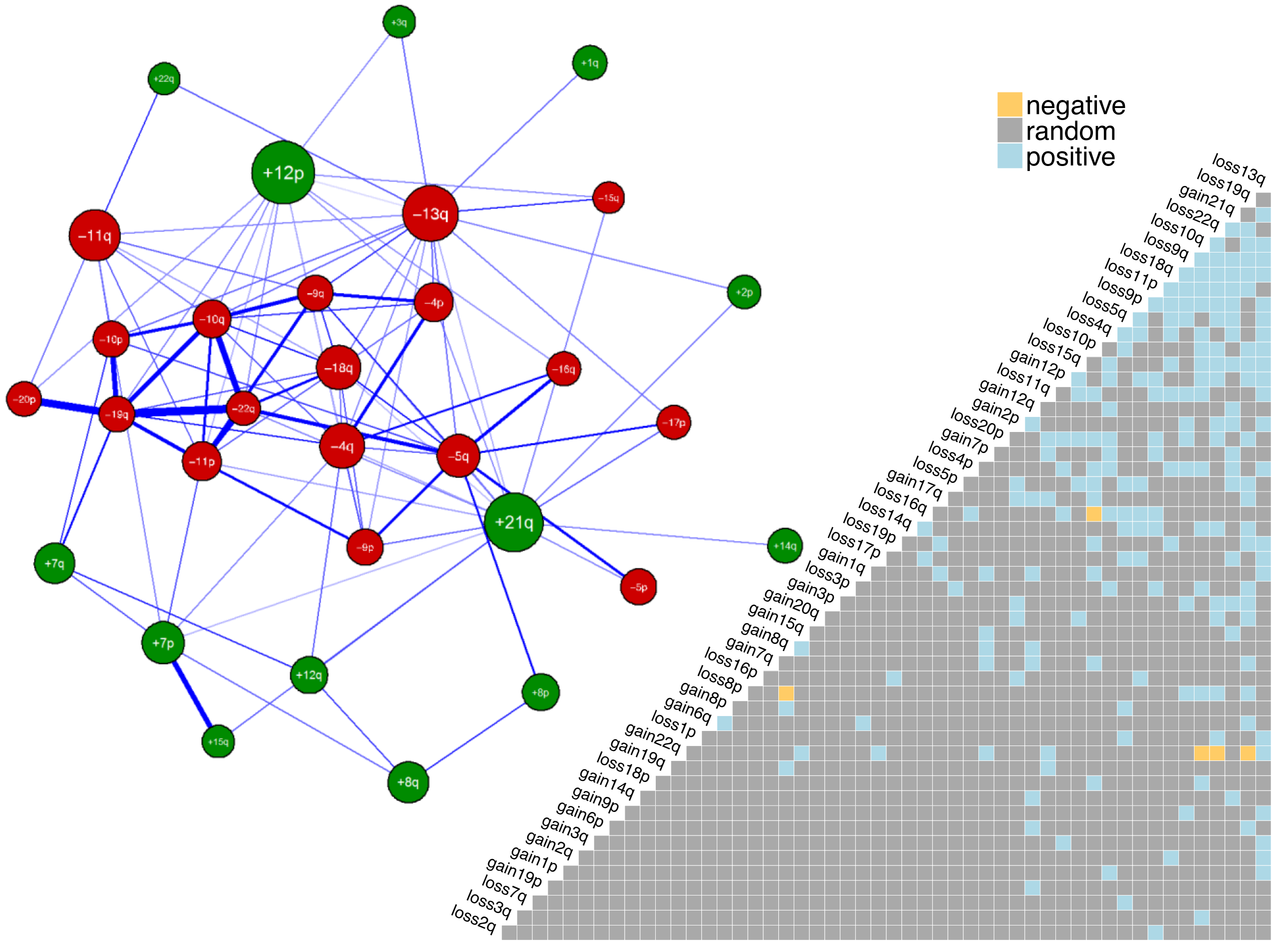

THCA (n=506; max p−value=0.00247)

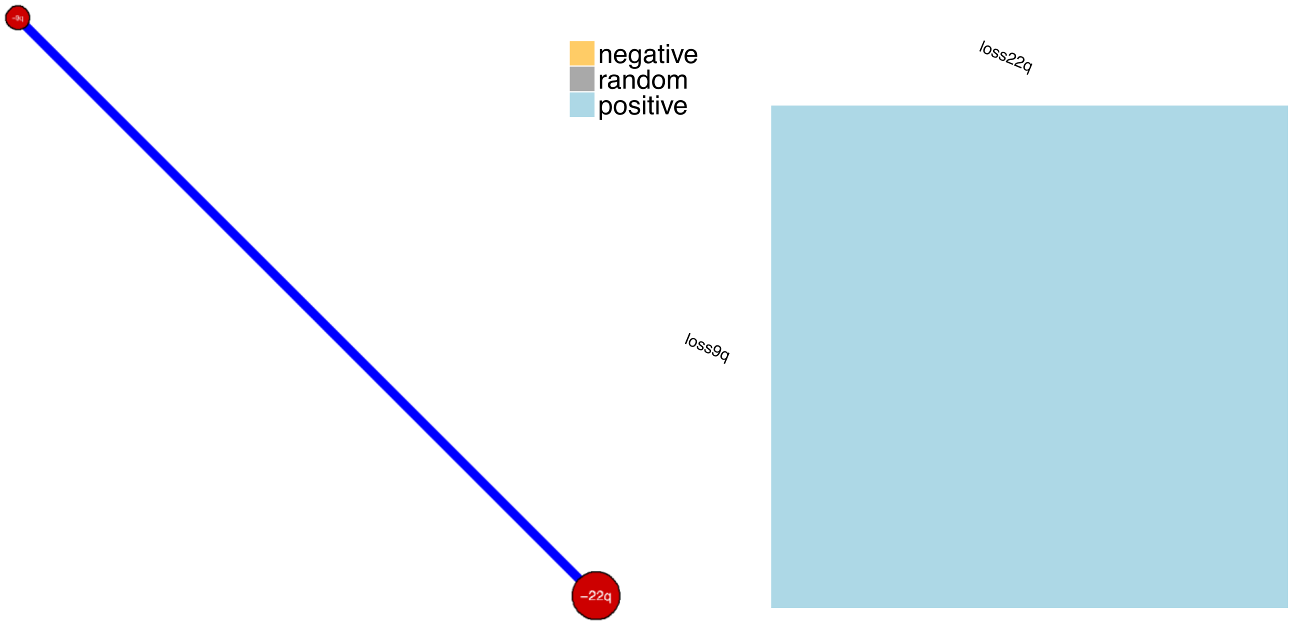

THYM (n=123; max p−value=0.00028)

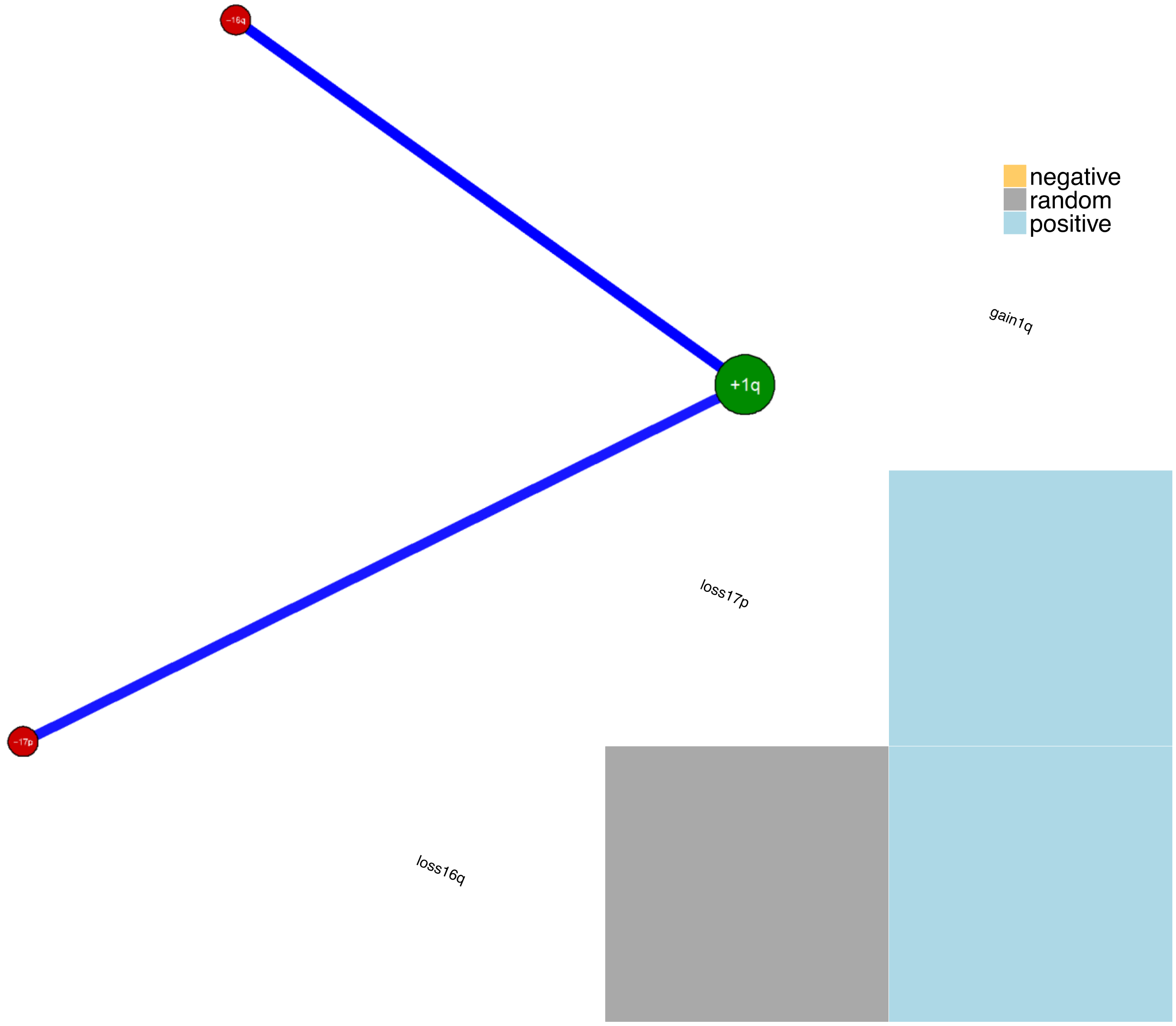

UCEC (n=544; max p−value=0.04922)

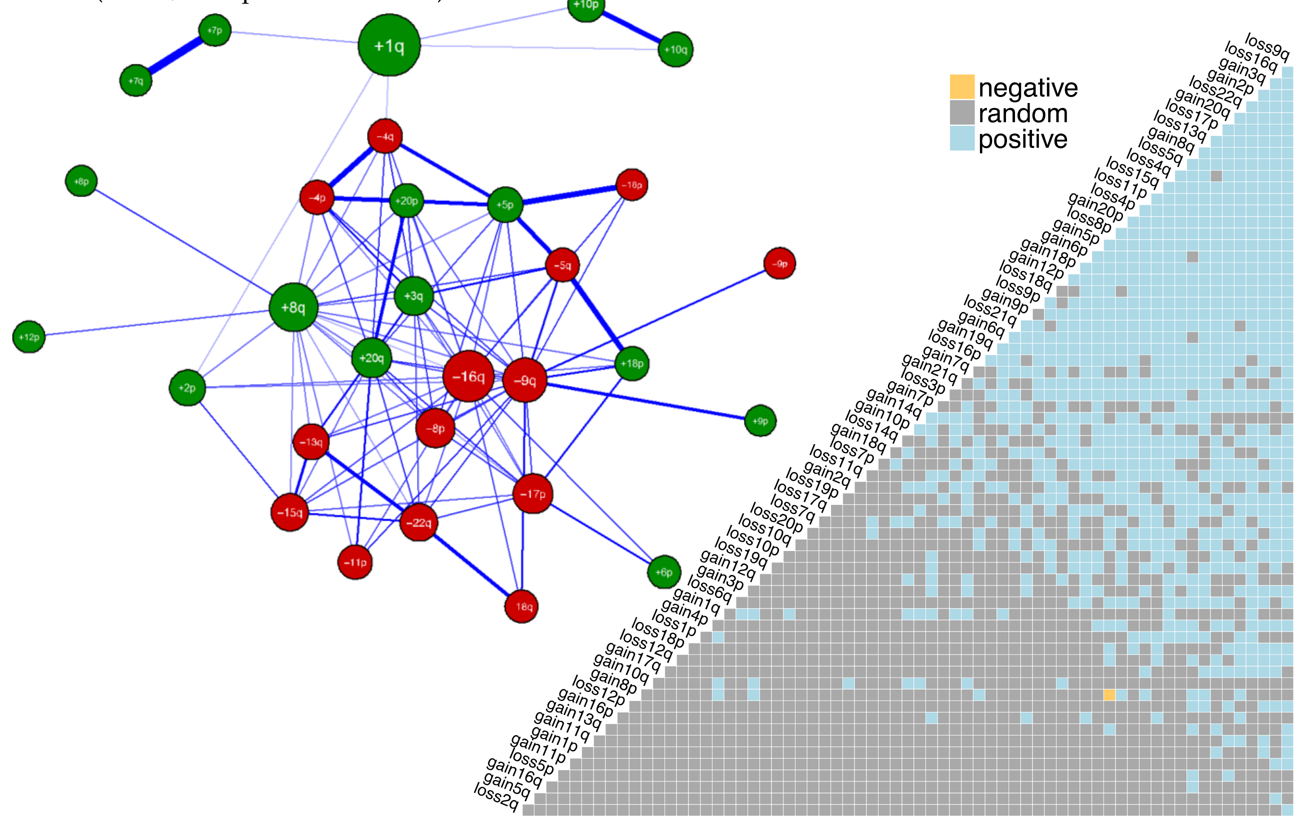

UCS (n=56; max p−value=0.0483)

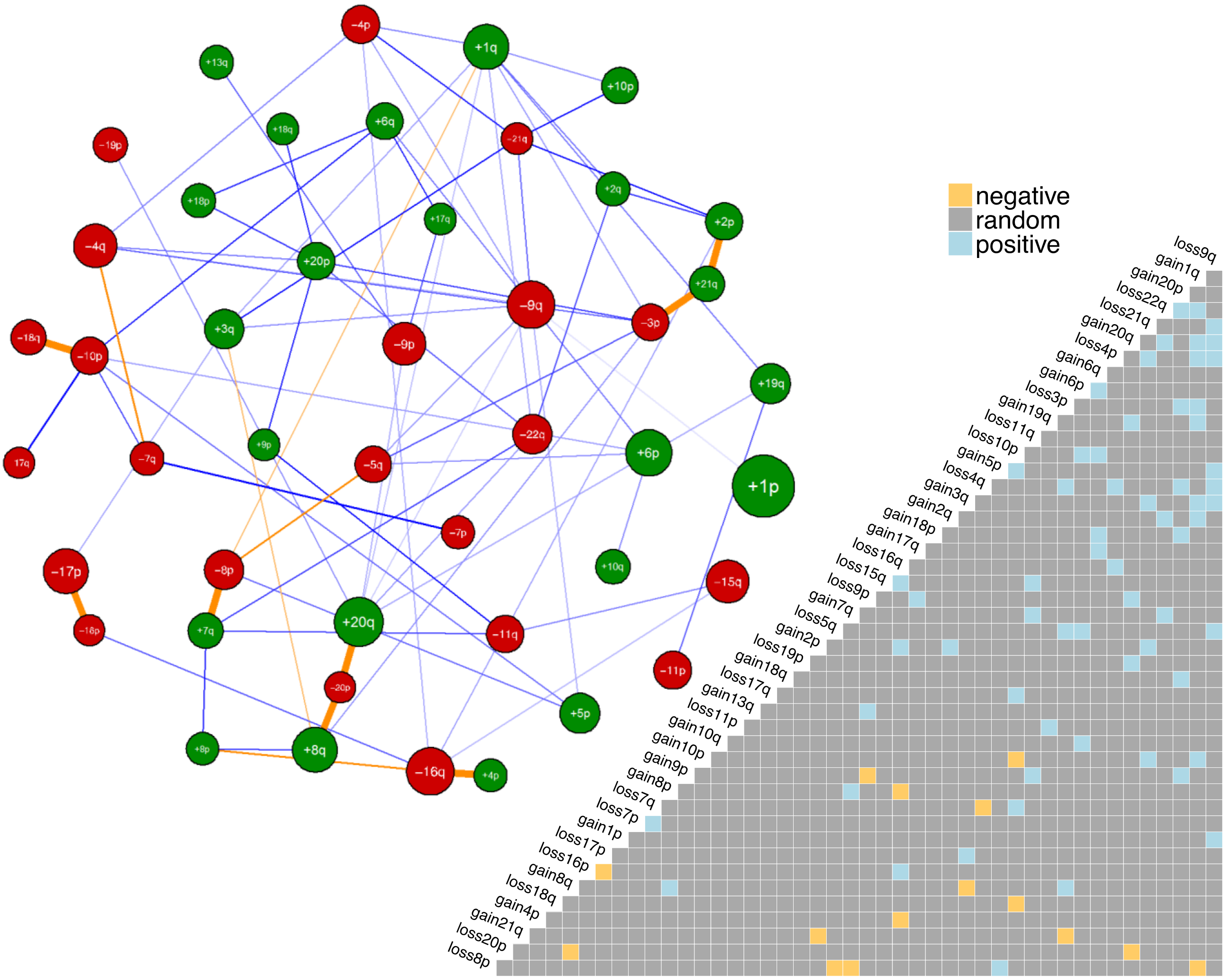

UVM (n=80; max p−value=0.04852)

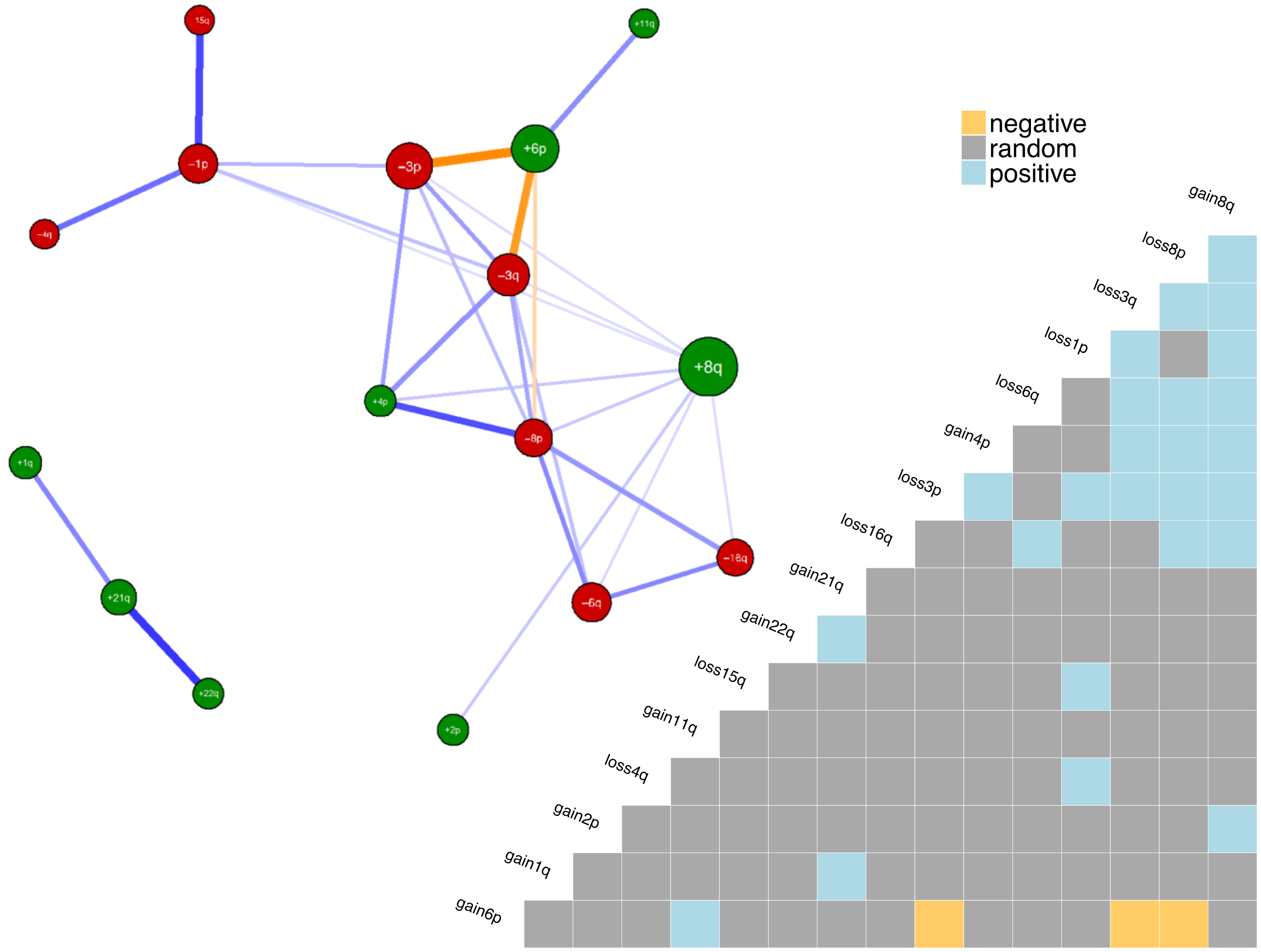

Supplementary Figure 9 (pages 12-27). Networks and matrices of CAA co-occurrences in 31 types of cancer For all cancer datasets, networks and matrices of CAA co-occurrences were built using a previously described probabilistic model, originally generated to identify statistically significant pair-wise patterns of species co-occurrence [3]. **Left:** CAA networks consist of nodes and edges. Nodes represent CAAs in green and red for chromosome arm-level gain or loss, respectively, and are size-proportional, i.e., CAAs that occur at higher frequencies are represented by larger size nodes. Connecting edges refer to CAA co-occurrences that occur at higher frequencies (positive associations; blue) or at lower frequencies (negative associations; orange) than expected by chance – while accounting for the frequencies of each of the two individual CAAs – and are also size-proportional, i.e., thicker edges represent lower *p* values. Shown are either all statistically significant associations ($p<0.05$), if there are ≤100, or the top 100 most statistically significant associations. Maximum *p* values are also shown. **Right:** Matrices of CAA co-occurrences for each cancer type. Boxes are colour-coded as per edge colours in the networks. Grey boxes represent non-statistically significant co-occurrence ($p>0.05$). CAAs that are not shown did not statistically significantly co-occur positively or negatively with any other CAA.

ACC

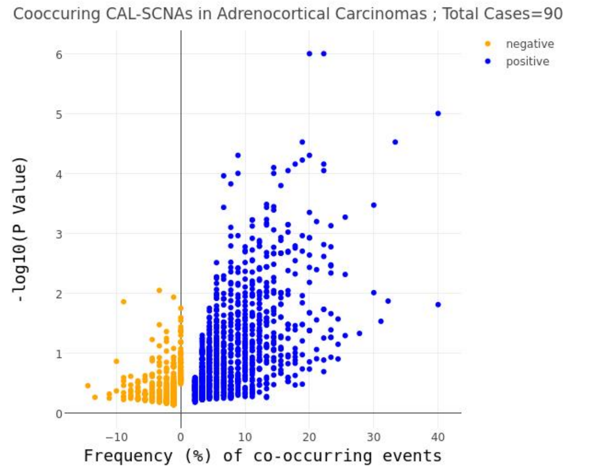

BLCA

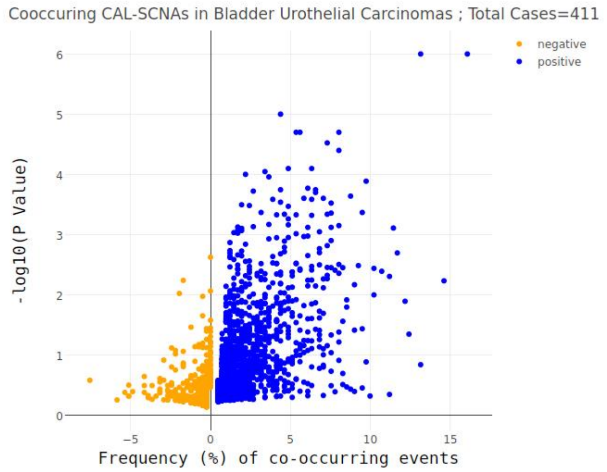

BRCA

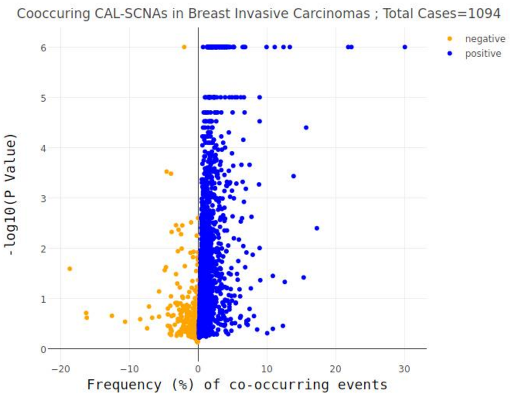

CESC

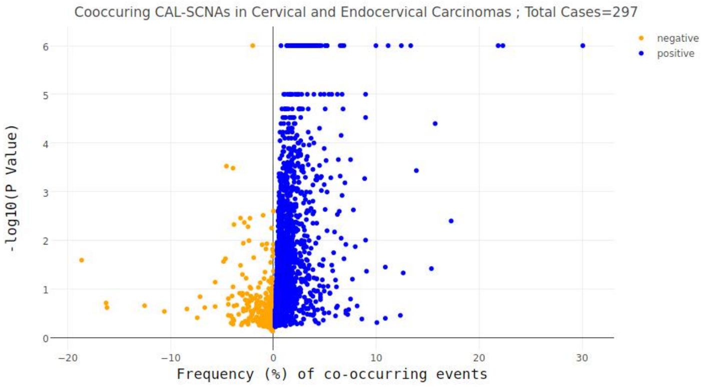

CHOL

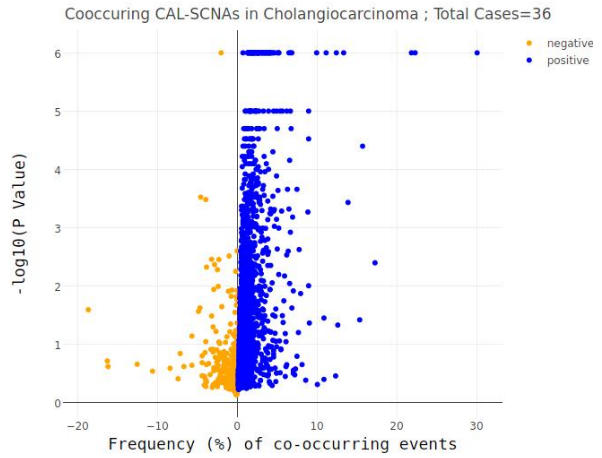

COADREAD

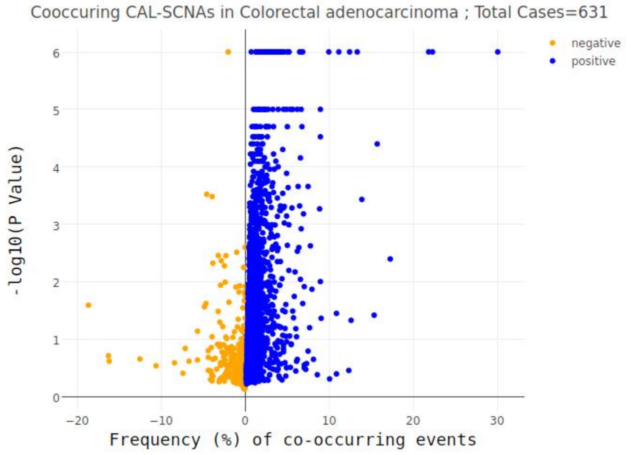

DLBCL

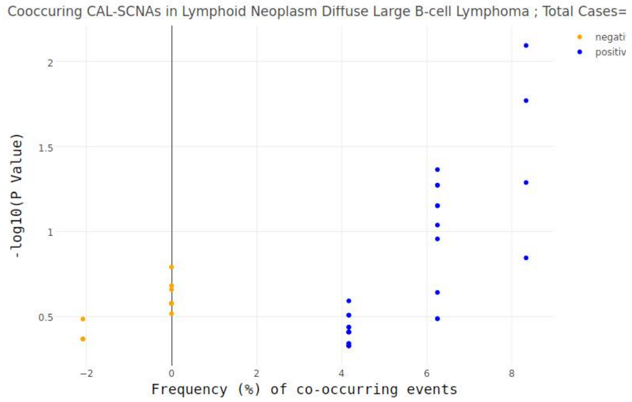

ESCA

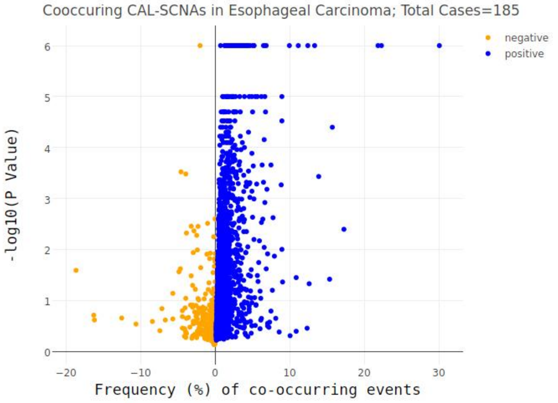

GBM

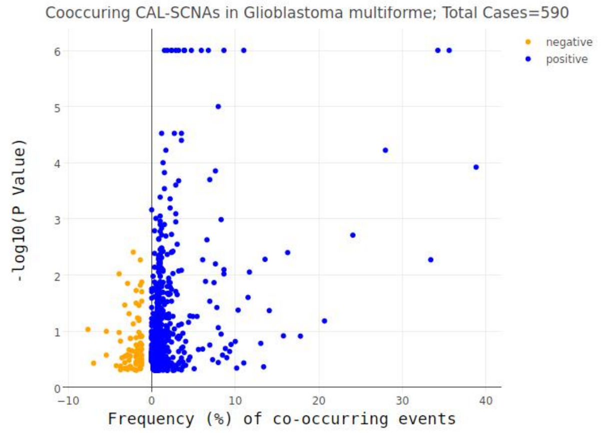

HNSC

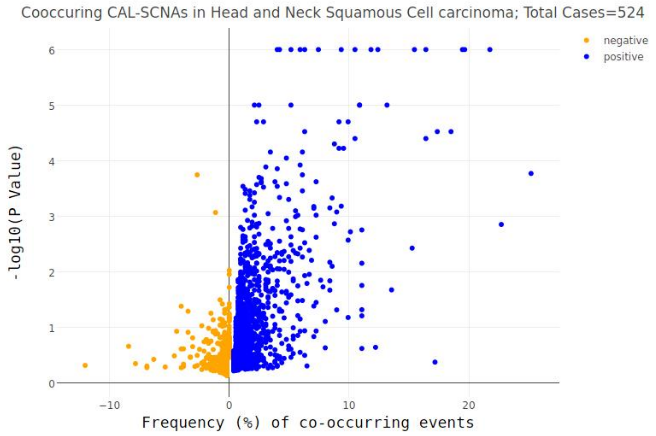

KIRC

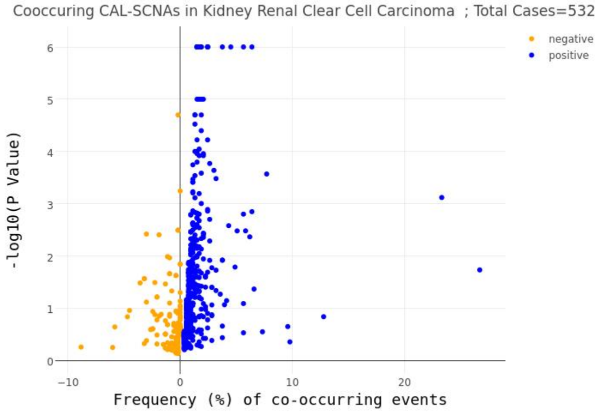

KIRP

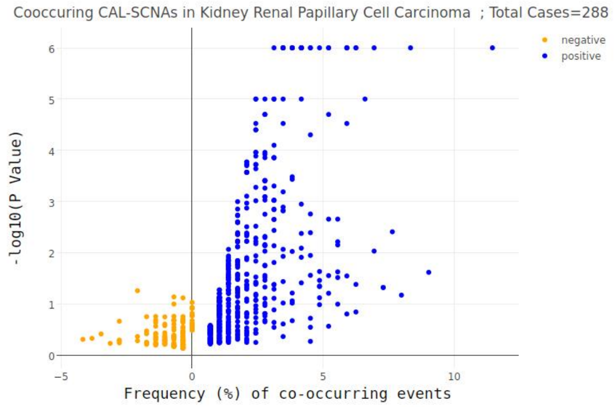

LAML

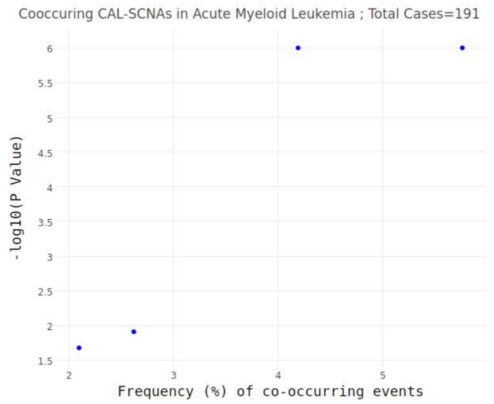

LGG

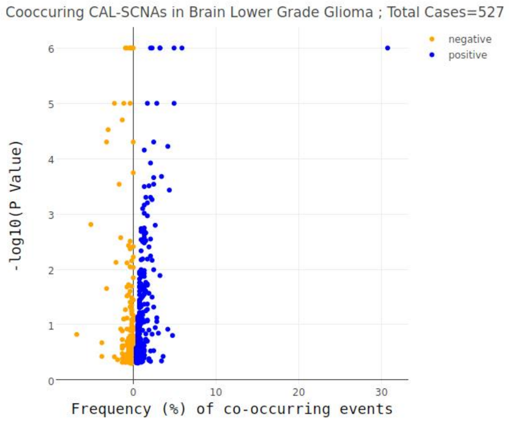

LIHC

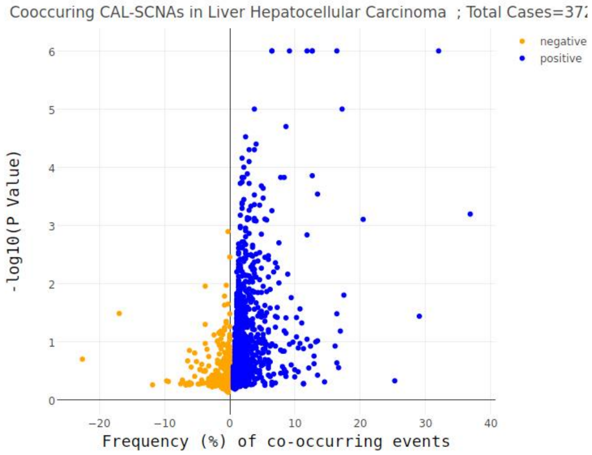

LUAD

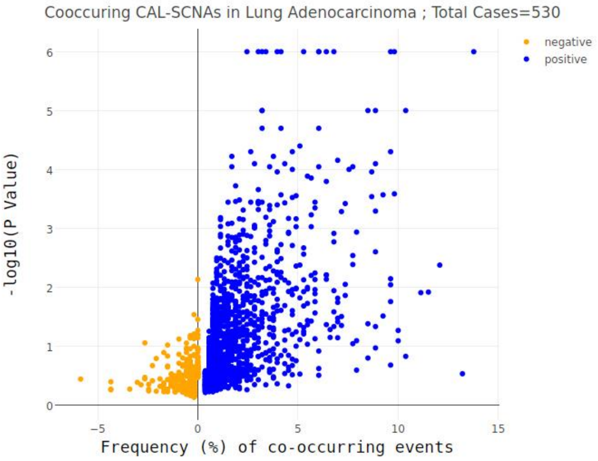

LUSC

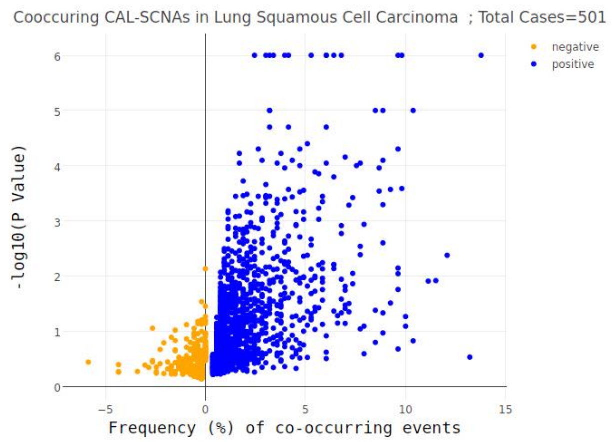

MESO

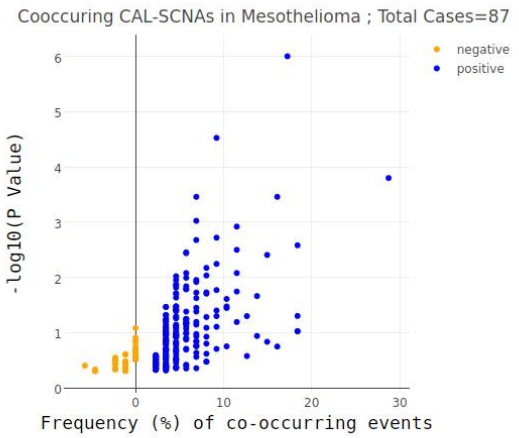

OV

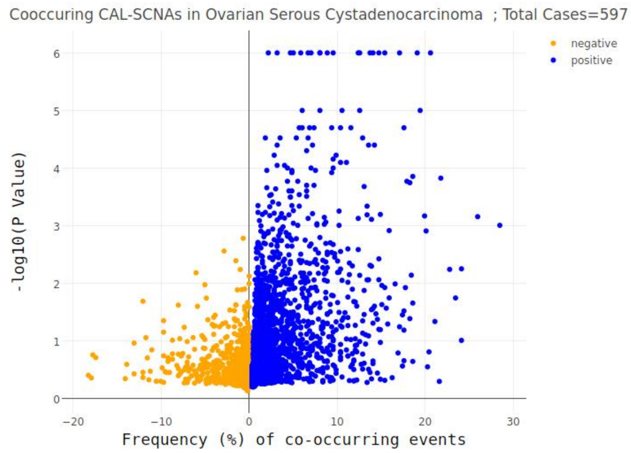

PAAD

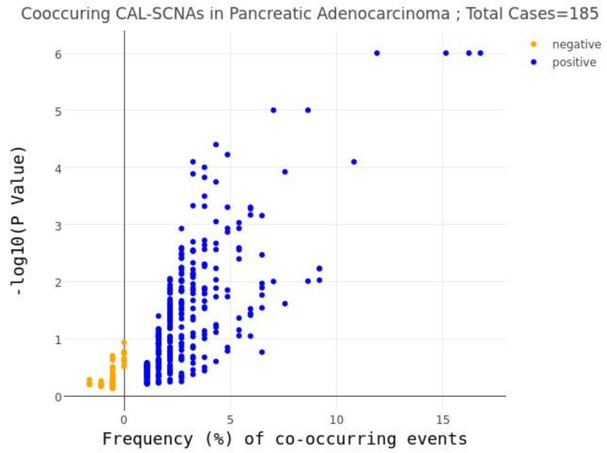

PCPG

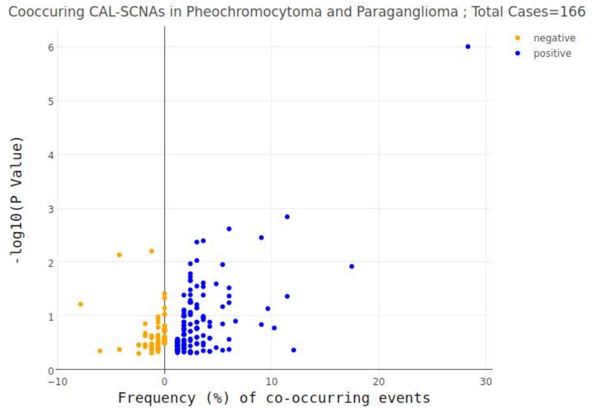

PRAD

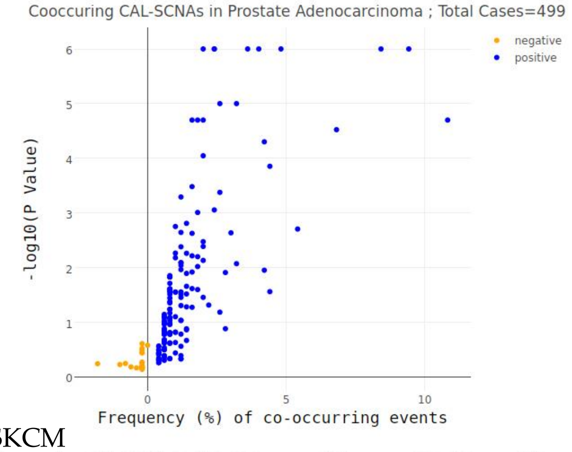

SARC

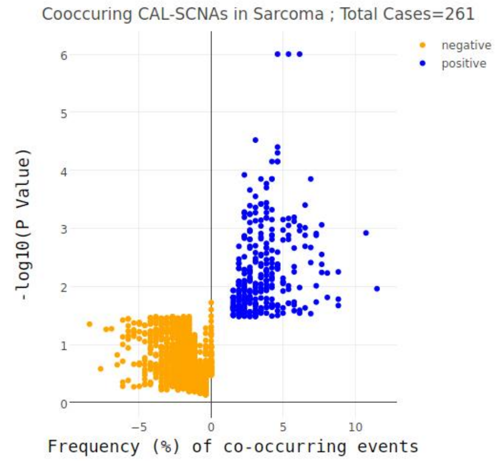

SKCM

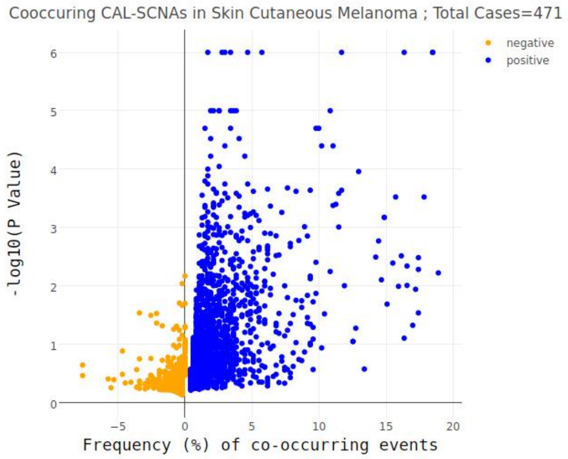

STAD

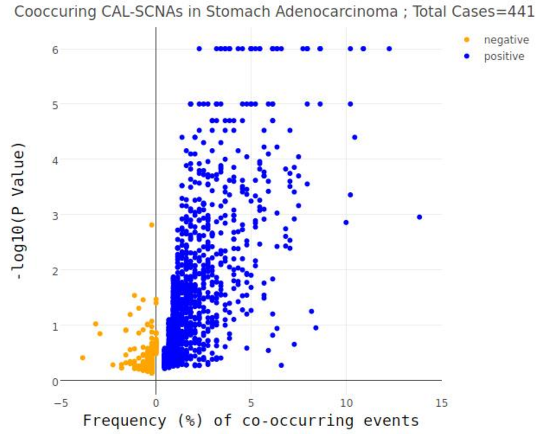

TGCT

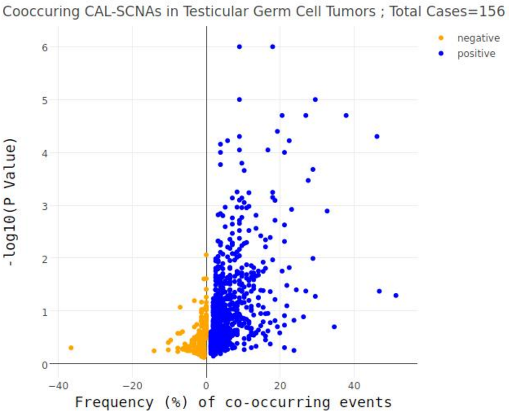

THCA

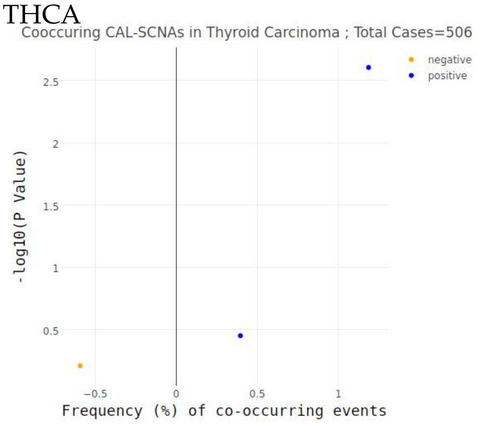

THYM

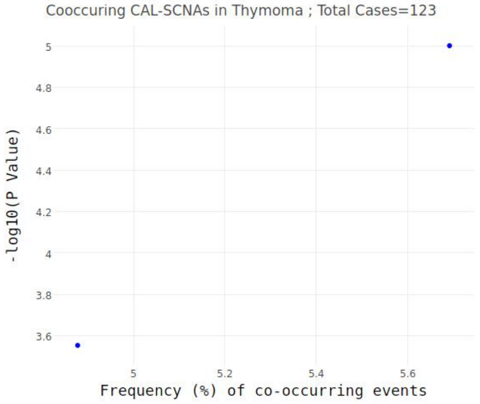

UCEC

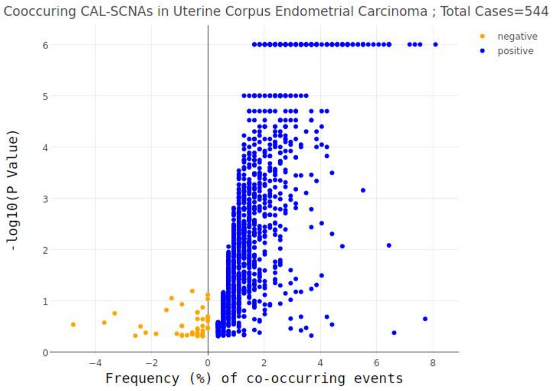

UCS

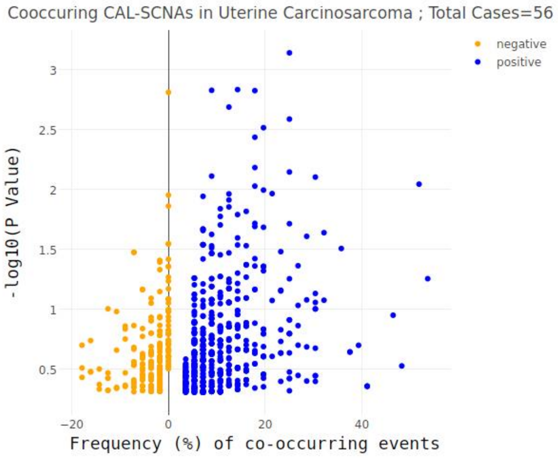

UVM

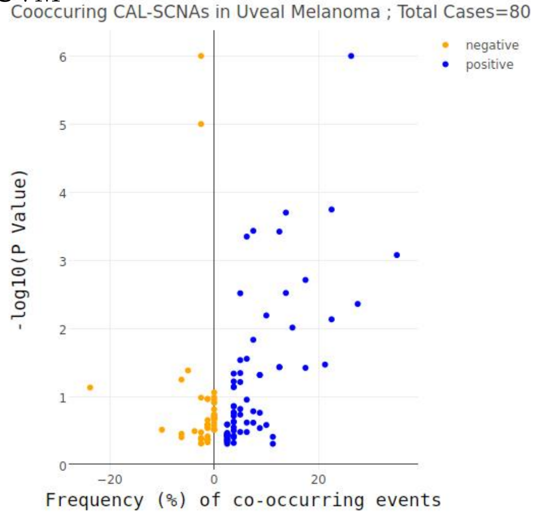

Supplementary Figure 10 (pages 28-31). Volcano plots of CAA co-occurrences in 31 types of cancer
For all cancer datasets, the frequencies of pairs of co-occurring CAAs (here called 'CAL-SCNAs') were plotted compared to their $p\ values$, as calculated according to a pairwise probabilistic cooccurrence model [3]. Note that where $-\log(p\ value) = 6$, this represents $p < 10^{-5}$, rather than $p = 10^{-6}$. 'Positive' cooccurrence (blue symbols) indicates cooccurrence at higher frequency than expected by chance. 'Negative' cooccurrence (orange symbols) indicates cooccurrence at lower frequency than expected by chance. See also Table S6.

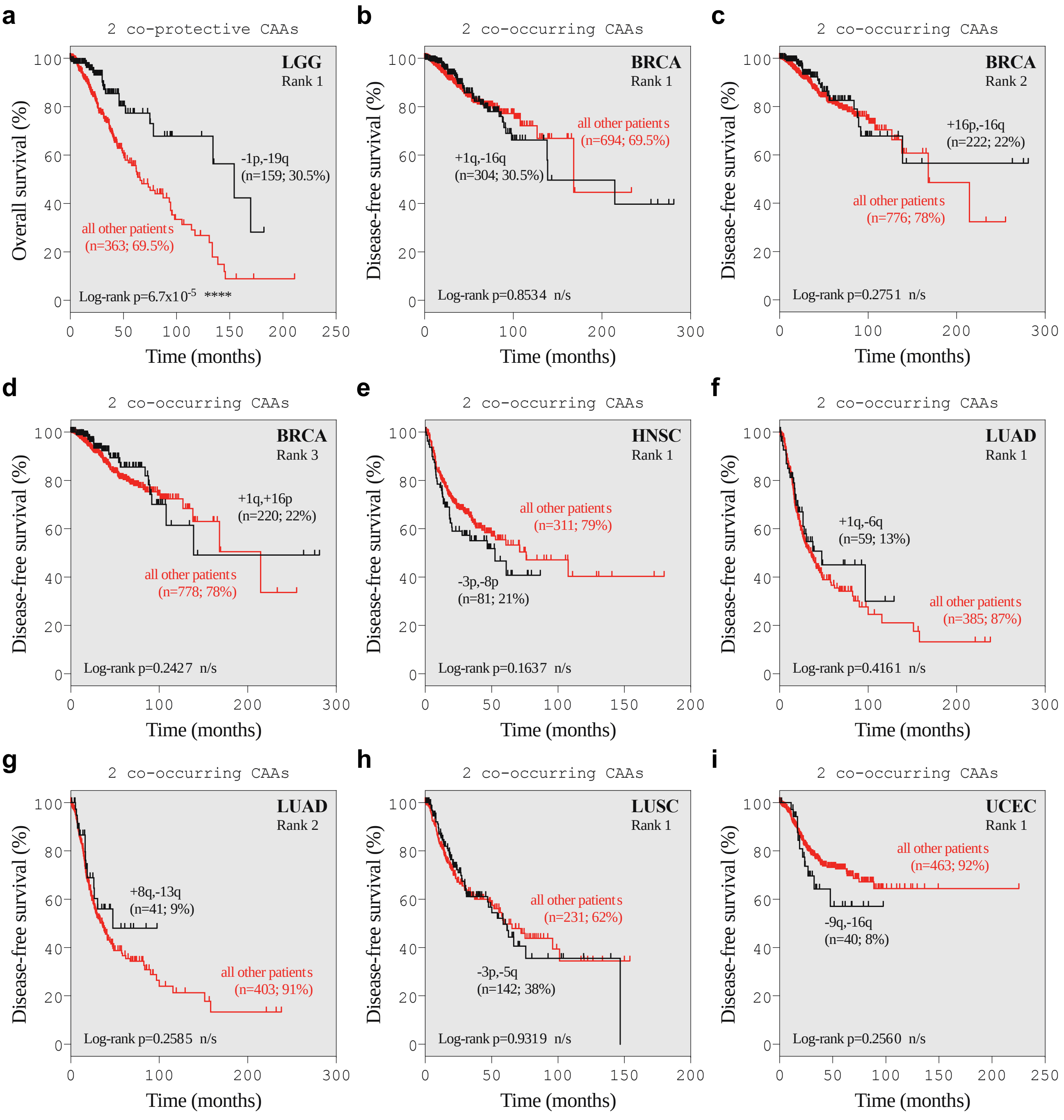

Supplementary Figure 11. Pan-cancer survival analysis based on significantly co-occurring CAAs
(a-i) Kaplan-Meier survival curves of top-ranking co-occurring CAAs following probabilistic co-occurrence analysis. *P* values: univariate log-rank tests.

a

CFE/CAA-drug interactions in 22 cancer types

p = 7.9×10^{-7}

	n			%		
	CFE	CAA	total	CFE	CAA	total
interaction	301	64	365	1.93	3.73	2.11
no interaction	15,319	1,652	16,971	98.07	96.27	97.89
total	15,620	1,716				

← 78 CAAs × 22 cancer types

710 CFEs × 22 cancer types

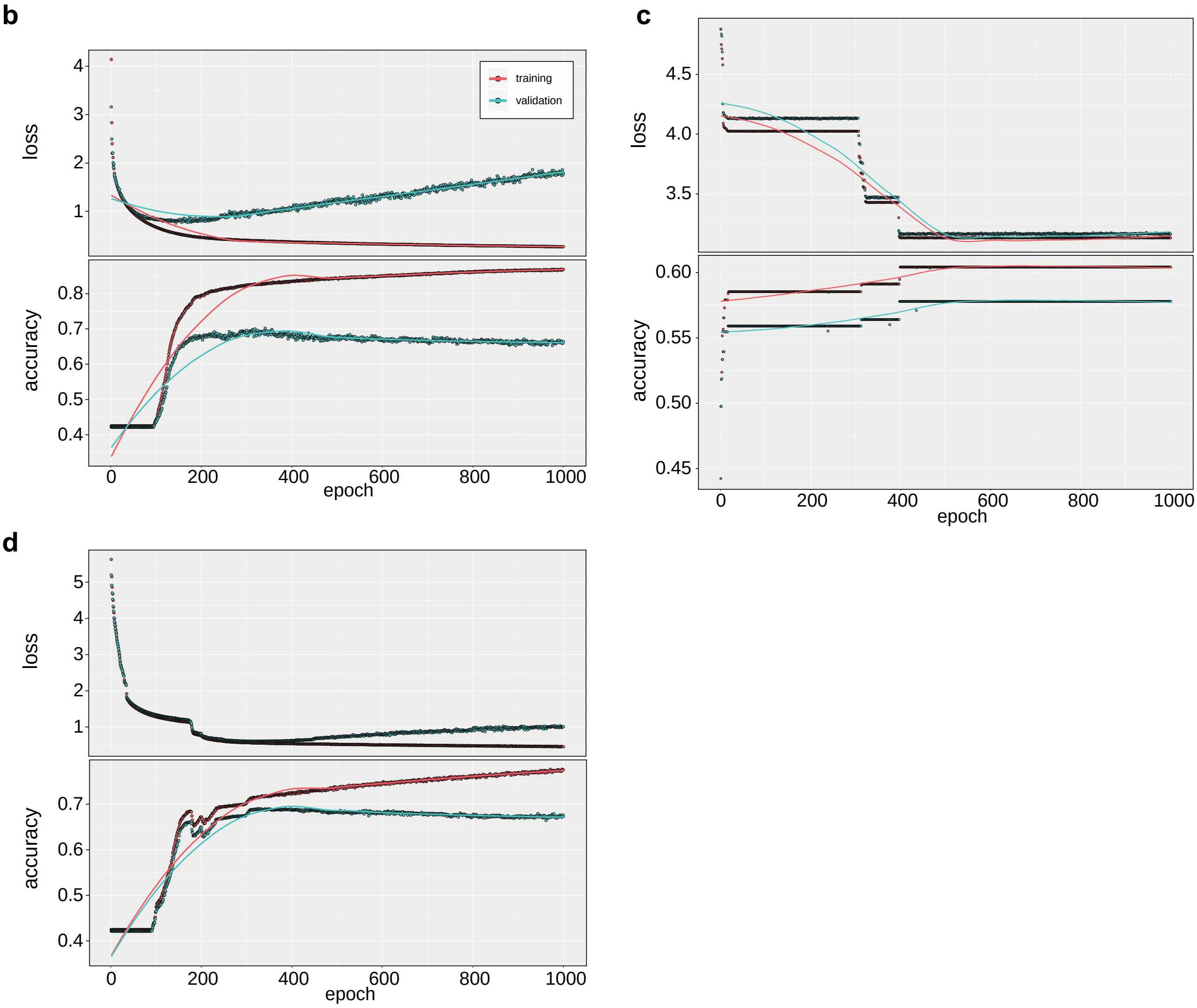

Supplementary Figure 12. Performance of CAA- and CFE-based machine learning models
(a) Summary table showing the relative involvement of CFEs and CAAs in pharmacogenomic interactions across 22 cancer types. **(b-d)** Loss and accuracy of deep learning models that include both cancer functional events (CFEs) and CAAs (b), only CFEs (c) or only CAAs (d), all as a function of the number of epochs, are shown for the training and validation sets.

Supplementary References

1. Taylor, A.M., et al., *Genomic and Functional Approaches to Understanding Cancer Aneuploidy.* Cancer Cell, 2018. **33**(4): p. 676-689 e3.
2. Curtis, C., et al., *The genomic and transcriptomic architecture of 2,000 breast tumours reveals novel subgroups.* Nature, 2012. **486**(7403): p. 346-52.
3. Veech, J.A., *A probabilistic model for analysing species co-occurrence.* Global Ecol Biogeogr, 2013. **22**: p. 252-60.
