# Supplementary material for: Chromosome arm aneuploidies shape tumour evolution and drug response
Source: Nat Commun. 2020 Jan 23;11:449. doi: 10.1038/s41467-020-14286-0 (PMC6978319; doi:10.1038/s41467-020-14286-0)
Supplement: Supplementary file 3 — Reporting Summary [file 41467_2020_14286_MOESM3_ESM.pdf]

nature research

Corresponding author(s): Pascal H.G. Duijf
Email: p.duijf@uq.edu.au

Last updated by author(s): Dec 2, 2019

Reporting Summary

Nature Research wishes to improve the reproducibility of the work that we publish. This form provides structure for consistency and transparency in reporting. For further information on Nature Research policies, see Authors & Referees and the Editorial Policy Checklist .

Statistics

For all statistical analyses, confirm that the following items are present in the figure legend, table legend, main text, or Methods section.

n/a	Confirmed	
☐	☒	The exact sample size (*n*) for each experimental group/condition, given as a discrete number and unit of measurement
☐	☒	A statement on whether measurements were taken from distinct samples or whether the same sample was measured repeatedly
☐	☒	The statistical test(s) used AND whether they are one- or two-sided<br>*Only common tests should be described solely by name; describe more complex techniques in the Methods section.*
☐	☒	A description of all covariates tested
☐	☒	A description of any assumptions or corrections, such as tests of normality and adjustment for multiple comparisons
☐	☒	A full description of the statistical parameters including central tendency (e.g. means) or other basic estimates (e.g. regression coefficient) AND variation (e.g. standard deviation) or associated estimates of uncertainty (e.g. confidence intervals)
☐	☒	For null hypothesis testing, the test statistic (e.g. *F*, *t*, *r*) with confidence intervals, effect sizes, degrees of freedom and *P* value noted<br>*Give P values as exact values whenever suitable.*
☒	☐	For Bayesian analysis, information on the choice of priors and Markov chain Monte Carlo settings
☒	☐	For hierarchical and complex designs, identification of the appropriate level for tests and full reporting of outcomes
☐	☒	Estimates of effect sizes (e.g. Cohen's *d*, Pearson's *r*), indicating how they were calculated

Our web collection on statistics for biologists contains articles on many of the points above.

Software and code

Policy information about availability of computer code

Data collection	No new data were collected in this study. The data used in this manuscript
Data analysis	Data were analyzed using Python (version 3), R (version 3.6) or GraphPad Prism 7 for Mac OS X (version 7.0e, September 5, 2018). The source code for the analyses in this study can be accessed via https://github.com/pascalduijf/CAAs_1.

For manuscripts utilizing custom algorithms or software that are central to the research but not yet described in published literature, software must be made available to editors/reviewers. We strongly encourage code deposition in a community repository (e.g. GitHub). See the Nature Research guidelines for submitting code & software for further information.

Data

Policy information about availability of data

All manuscripts must include a data availability statement. This statement should provide the following information, where applicable:

- Accession codes, unique identifiers, or web links for publicly available datasets
- A list of figures that have associated raw data
- A description of any restrictions on data availability

Publicly available datasets used in this study, and their accession information, are: TCGA: GDC Data Portal https://portal.gdc.cancer.gov (unrestricted public access for the data used in this study); MSK-IMPACT: http://cbioportal.org/msk-impact (unrestricted public access); METABRIC: https://ega-archive.org (accession number EGAD00010000164; restricted access); GDSC: https://ega-archive.org (accession number EGAS00001000978; unrestricted access). Raw data are provided in the Source Data file and Supplementary Tables. Further details are provided in the Methods section entitled Data Sources.

Field-specific reporting

Please select the one below that is the best fit for your research. If you are not sure, read the appropriate sections before making your selection.

- [x] Life sciences
- [ ] Behavioural & social sciences
- [ ] Ecological, evolutionary & environmental sciences

For a reference copy of the document with all sections, see nature.com/documents/nr-reporting-summary-flat.pdf

Life sciences study design

All studies must disclose on these points even when the disclosure is negative.

Sample size	Sample sizes were not predetermined. All available samples were included in the analyses and no samples were omitted.
Data exclusions	No data were excluded from the analyses. Where sample sizes of subgroups do not add up to the total sample size, this was due to missing information. For example, patient survival data are not available for each patient. In such cases, all patients with available data were included.
Replication	Replication and validation occurred on several levels. Methodological and biological validation occurred using independent methods and raw data types (SNP6 array copy number data, whole-genome array data and whole-genome sequencing data) and independent patient cohorts (TCGA, ICGC, MSK-IMPACT and METABRIC). In addition, machine learning models employed cross-validation (see also below and Methods section).
Randomization	Our machine learning approach involved splitting of samples into groups. This occurred at random. To minimize over-fitting and potential artifacts due to over- or under-representation of subgroups within the randomly split groups, which could have occurred by coincidence, we performed multiple rounds of cross-validation. Specifically, our model was trained on 80% of the data. The remaining 20% served as test data. To avoid over-fitting, the training data were randomly split into 5 equal parts to enable performing 5 times 5-fold cross-validation on the data within the training set. The performance measure used was the average of the values computed on the 5 models. Finally, the model learned was validated on the test data.
Blinding	Group allocation, as described in the section above, occurred computationally and at random. Investigators were blinded to this group allocation.

Reporting for specific materials, systems and methods

We require information from authors about some types of materials, experimental systems and methods used in many studies. Here, indicate whether each material, system or method listed is relevant to your study. If you are not sure if a list item applies to your research, read the appropriate section before selecting a response.

Materials & experimental systems

n/a	Involved in the study	
☒	☐	Antibodies
☒	☐	Eukaryotic cell lines
☒	☐	Palaeontology
☒	☐	Animals and other organisms
☒	☐	Human research participants
☒	☐	Clinical data

Methods

n/a	Involved in the study	
☒	☐	ChIP-seq
☒	☐	Flow cytometry
☒	☐	MRI-based neuroimaging
