# Supplementary material for: Chromosome arm aneuploidies shape tumour evolution and drug response
Source: Nat Commun. 2020 Jan 23;11:449. doi: 10.1038/s41467-020-14286-0 (PMC6978319; doi:10.1038/s41467-020-14286-0)
Supplement: Supplementary file 4 — Description of Additional Supplementary Files [file 41467_2020_14286_MOESM4_ESM.pdf]

Description of Additional Supplementary Files

File name: Supplementary Data 1
Description: The Cancer Genome Atlas (TCGA) patient cohorts and cohort groups used in this study

File name: Supplementary Data 2
Description: Primary and metastatic samples of the MSK-IMPACT cohort used in this study

File name: Supplementary Data 3
Description: CAA transition probabilities in the TCGA-BRCA dataset

File name: Supplementary Data 4
Description: Single and co-occurring CAAs that predict good or poor overall survival

File name: Supplementary Data 5
Description: Single and co-occurring CAAs that predict good or poor disease-free survival

File name: Supplementary Data 6
Description: Co-occurring CAAs across 31 cancer types

File name: Supplementary Data 7
Description: Summary table of single and co-occurring CAAs that predict good or poor patient survival

File name: Supplementary Data 8
Description: Correlation between CAA burden and predicted pCR to preoperative T/FAC chemotherapy

File name: Supplementary Data 9
Description: Members of the TFAC pharmaco-genomic gene expression predictor

File name: Supplementary Data 10
Description: Pan-cancer features predicting increased drug resistance or sensitivity using the expanded GDSC dataset

File name: Supplementary Data 11
Description: Identification of potential synthetic lethal and synergistic resistance pharmacogenomic interactions

File name: Supplementary Data 12
Description: Pharmacogenomic interactions of co-lost and co-gained focal copy number alterations on the same chromosome arm

File name: Supplementary Data 13
Description: CAA and CFE model performance per drug

File name: Supplementary Data 14
Description: Centromere regions excluded from CAA analyses
